# The Bony Nasal Cavity and Paranasal Sinuses of Big Felids and Domestic Cat: A Study Using Anatomical Techniques, Computed Tomographic Images Reconstructed in Maximum-Intensity Projection, Volume Rendering and 3D Printing Models

**DOI:** 10.3390/ani14172609

**Published:** 2024-09-07

**Authors:** Elena Díaz Martínez, Alberto Arencibia Espinosa, Marta Soler Laguía, María Dolores Ayala Florenciano, David Kilroy, María I. García García, Francisco Martínez Gomariz, Cayetano Sánchez Collado, Francisco Gil Cano, José Raduán Jaber, Gregorio Ramírez Zarzosa

**Affiliations:** 1 Department of Anatomy and Comparative Pathological Anatomy, Veterinary Faculty, University of Murcia, 30100 Murcia, Spain; elena.diaz2@um.es (E.D.M.); mdayala@um.es (M.D.A.F.); f.gomariz@colvet.es (F.M.G.); scollado@um.es (C.S.C.); cano@um.es (F.G.C.); 2 Department of Morphology, Anatomy and Embriology, Veterinary Faculty, University of Las Palmas de Gran Canaria, Transmontaña, Arucas, 35416 Las Palmas, Spain; alberto.arencibia@ulpgc.es (A.A.E.); joseraduan.jaber@ulpgc.es (J.R.J.); 3 Department of Medicine and Surgery, Veterinary Faculty, University of Murcia, 30100 Murcia, Spain; mtasoler@um.es; 4 UCD Veterinary Science Centre, University College Dublin, D04 V1W8 Dublin, Ireland; david.kilroy@ucd.ie; 5 Support Research Service SACE-ACTI, University of Murcia, 30100 Murcia, Spain; mariagarcia@um.es

**Keywords:** trepanation, skull, computed tomography, image techniques, reconstruction, three-dimensional printing, osseous nasal cavity, feline, felidae, anatomy

## Abstract

**Simple Summary:**

To date, there are very few studies of the bony nasal cavity of the domestic cat and even fewer of big felids. A detailed and exhaustive anatomical study of this cavity in the domestic cat is presented in this paper through dissections, trepanations and anatomical sections. This work has allowed to delve deeper into the anatomy of the bony nasal cavity. This study deepens the knowledge of this region through diagnostic imaging techniques, such as computed tomography, three-dimensional reconstruction in maximum-intensity projection and volume rendering, that allow for the spatial visualization of this cavity. These reconstructed images represent an improvement in the anatomical study of the cavity and will help veterinary clinicians in the diagnosis of nasal pathologies. Obtaining molds by three-dimensional printing allows for acquiring models very close to reality that capture these structures in detail. These techniques, which in human medicine have already led to many clinical and therapeutic improvements, are a very useful tool in veterinary medicine that will lead to advances and improvements in the diagnosis and treatment of nasal pathologies, improving the survival of many threatened species.

**Abstract:**

This study aims to develop three-dimensional printing models of the bony nasal cavity and paranasal sinuses of big and domestic cats using reconstructed computed tomographic images. This work included an exhaustive study of the osseous nasal anatomy of the domestic cat carried out through dissections, bone trepanations and sectional anatomy. With the use of OsiriX viewer, the DICOM images were postprocessed to obtaining maximum-intensity projection and volume-rendering reconstructions, which allowed for the visualization of the nasal cavity structures and the paranasal sinuses, providing an improvement in the future anatomical studies and diagnosis of pathologies. DICOM images were also processed with AMIRA software to obtain three-dimensional images using semiautomatic segmentation application. These images were then exported using 3D Slicer software for three-dimensional printing. Molds were printed with the Stratasys 3D printer. In human medicine, three-dimensional printing is already of great importance in the clinical field; however, it has not yet been implemented in veterinary medicine and is a technique that will, in the future, in addition to facilitating the anatomical study and diagnosis of diseases, allow for the development of implants that will improve the treatment of pathologies and the survival of big felids.

## 1. Introduction

The anatomical study of the bones and cartilages of the nose and nasal cavity is essential to understanding the supportive structure of this cavity. The nose is restricted to the vertex and more specifically to the nostrils, and the openings to the nasal cavity. The nostrils remain open thanks to the cartilaginous framework that supports the external opening of the nose. These cartilages are attached to the nasal septum cartilage and covered dorsally by the nasal bone, ventrally by the incisive bone and laterally by the maxilla. In this context, it is worth highlighting the dorsal lateral nasal cartilage, which forms the anatomical base of the dorsum of the openings. On the other hand, the ventral lateral nasal cartilage is another important structure in this conformation and develops from the ventral edge of the nasal septum. These two cartilages are accompanied by two accessory nasal cartilages: the lateral and the medial accessory nasal cartilages. Finally, the cartilaginous base of the wing of the nose resides in the alar cartilage [1]. Díaz Martínez et al. [2] described the external nose of felids using computed tomography (CT), magnetic resonance imaging (MRI) and rhinoscopy. In our study, we also described it through dissections, trepanations, anatomical sections and three-dimensional (3D) reconstructions of CT images. The nasal vestibule is the beginning of the nasal cavity. The straight, alar and basal folds, of cartilaginous nature, are rostral extensions of the dorsal and ventral conchae, respectively, that fix and support the nasal vestibule [1].

The nasal cavity is made up of meatuses, openings, paranasal sinuses and ethmoturbinates (ectoturbinates and endoturbinates), which are attached to the ethmoid bone [1]. The roof of the nasal cavity is formed by the nasal bone and part of the frontal bone; laterally it is limited by the frontal, lacrimal, maxillary, premaxillary (incisive) and palatine bones; and at the ventral level by the horizonal plates of the palatine, maxillary and premaxillas (incisive bones) [3]. Among the ethmoturbinates, it should be noted that the dorsal nasal concha is a simple sheet that originates from the ethmoid bone and is also attached rostrally to the nasal bone. On the other hand, the ventral nasal concha is thick and short and originates from the maxilla and separates from it to roll on itself, giving rise to a complex structure that considerably increases the surface of the highly vascularized nasal cavity [4]. These structures, covered with mucosa, have a bony base made up of fine and thin sheets that support them [5]. It is worth highlighting in this context the poor development of the dorsal and ventral nasal conchae in felids and the great development of the third endoturbinate. To date, no studies have been published that describe with very detailed images the bony base of the feline nasal cavity, and with it the extensions and outgrowths of the conchal and ethmoturbinate bones. Only atlases and anatomical treatises such as those of Crouch [6], Hudson et al. [7] and Reighard et al. [3] described the bone conformation of the cat nasal cavity and detailed the structures that make it up. In addition to these treatises, Konig [8] carried out a detailed study of the nasal cavity using anatomical techniques, drawings and diagrams. Konig et al. [9] offered an expansion and update of the latter based on a descriptive anatomy without the use of images.

The paranasal sinuses of carnivores are diverticula of the nasal cavity that extend internally within specific bones of the skull. These sinuses have different conformations depending on the species studied and are important for heating and humidifying the air [1]. The paranasal sinuses of the cat are the frontal and the sphenoidal sinuses and the maxillary recess, with the frontal sinus being the most developed. In the cat, the frontal sinus is single and is not divided into compartments as it is in the dog, and communication with the nasal cavity is in the rostral part of the sinus [4]. In the dog, there is a frontal sinus described as rostral, lateral, medial and a maxillary recess, and no sphenoidal and palatine sinuses are identified [1,10]. The sphenoidal sinus has been described in the cat [2,7,11]. The paranasal sinuses in horses, ruminants and pigs are considerably larger and more complex than in dogs and cats. The paranasal sinuses can be affected by various pathologies such as tumors, abscesses or fungal rhinitis and even foreign bodies [5,12,13,14,15,16].

Knowledge of the bony anatomy of the nasal cavity and paranasal sinuses of felids is essential for a correct understanding of their physiology, as well as the diagnosis of pathologies that may affect this region. The use of dissections, trepanations and sectional anatomy allows for the direct visualization of the bony nasal cavity and paranasal sinuses, which is the topic of this study. There are only a few publications of morphometric studies of the feline skull [17,18]. In this article we used the domestic cat as a type species, carefully describing the anatomy of the bony nasal cavity and paranasal sinuses using these techniques and then comparing our findings with some of the big felids in possible danger of extinction.

Modern diagnostic imaging techniques allow for the creation of 3D representations that provide high-quality diagnostic images for better evaluation and treatment of different animal diseases [19]. Imaging techniques that have been used to study the nasal cavity include CT and MRI. Studies by Losonsky et al. [20], Conchou et al. [11] and Díaz Martínez et al. [2] described the nasal cavity in the cat with these techniques. However, in our study, only CT was chosen as it permits a more detailed study of the bones and paranasal sinuses through 3D volumetric reconstructions of the bony nasal cavity of felids. Volumetric reconstruction using data acquired by CT with the OsiriX viewer improves the understanding of anatomy at this level, being considered as an auxiliary method for the description and diagnosis of pathologies [21]. With this same software, maximum-intensity projection (MIP) and volume-rendering (VR) images were obtained that allowed us to closely study the extension and size of the internal structures. To date, VR has only been used in cats to study the abdominal and pelvic cavity [22]. In dogs, this technique has been used to study the temporomandibular joint [21]. In exotic animals, there is only one study of the skull of the loggerhead sea turtle and the green iguana using this imaging technique [19]. It should be noted that, in big felids, due to the scarcity of available specimens, 3D reconstructions are a key technique to obtain more information about these species.

In addition, through these reconstructions, a 3D impression of the bony nasal cavity and paranasal sinuses of felids was also performed. We obtained physical molds of the bony nasal cavity of the domestic cat and some big felids, facilitating their visualization and analysis. To date, there are no anatomical studies in veterinary medicine using 3D impression models of the skull, nasal cavity or paranasal sinuses. In veterinary medicine, only Carvalho et al. [23] have used printed models of dog teeth to evaluate endodontic techniques. In human medicine, 3D impression models are increasingly used and have proven to be a suitable and rapid method to replicate structures of the nasal cavity and experimentally evaluate nasal function [24]. Furthermore, it is very useful in the volumetric evaluation of bone defects and has shown great accuracy in prototyping for surgical planning [25] and in the development of nasal implants for craniofacial reconstructions [26], as well as in the creation of endoscopic models of the nasal cavity for study [27], and in the development of nasal stents for patients with cleft lip [28].

In this article, CT was used to study the feline bony nasal cavity, after previously having made a thorough analysis of the region through dissections, trepanations and sectional anatomy. MIP and VR allowed us to create very valuable 3D images for the anatomical study of this cavity. Furthermore, the creation of 3D impression models represents an advance that allows for the replication of bony structures, and this will improve anatomical knowledge, the diagnosis of pathologies, surgical planning, as well as the manufacture of prostheses and implants.

## 2. Materials and Methods

### 2.1. Animals

For this study, a total of sixteen specimens were used, eleven of which were domestic cats (*Felis silvestris catus* L.) and five were big felids: one leopard (*Phantera pardus kotiya* L.), two lions (*Phantera leo leo* L.) and two cheetahs (*Acinonyx jubatus jubatus* S). Of the eleven domestic cats used, nine were cadavers and two were live cats. Seven cat cadavers were donated by the Zoonoses and Pest Control Service of Murcia and were euthanized for reasons unrelated to our study, and two cadavers were obtained from the Necropsy Room of the Department of Comparative and Pathological Anatomy of the University of Murcia. The two live cats were loaned to this study with prior consent from the owners (they were informed about the study and signed the inform consent). All domestic cats were aged between 2 and 3 years with an average weight of 2.5–3.5 kg, and there were four females and five males. 

Three big felids (a leopard, a lion and a cheetah) were obtained from the Terra Natura natural park (Murcia, Spain) and were euthanized for reasons beyond our study. The other two big felids (a lion and a cheetah) had died in zoos and circuses during the decades of 1980 to 2000 and these cadavers were obtained from the Necropsy Room of the Department of Comparative and Pathological Anatomy of the University of Murcia, Spain. This experiment was supervised by the Ethics Commission of the University of Murcia, Spain (REGAES300305440012 CEEA: 305/2017; extended on 25 July 2022 as project Type II).

### 2.2. Anatomical Techniques

#### 2.2.1. Dissections

A cat cadaver from the Pest Control Service of Murcia was injected with blue latex (NV001, Ballons CP, Espinardo, Murcia, Spain) through the external jugular veins. Subsequently, it was fixed by immersion in embalming solution (10% formaldehyde (1.5 L), glycerine (3 L), methyl alcohol (12.5 L), and phenol (1 L) for 7 days under refrigeration. The feline head was sectioned at the level of the neck using a band saw. Next, the head was cut in the sagittal plane and the head muscles, turbinates and conchae were dissected. 

#### 2.2.2. Trepanations

The two cat cadavers from the Necropsy Room were used to obtain two crania. A complete cat skull was trephined using a milling machine with a mini saw that cut the orbital surface of the frontal bone and the wings of the basisphenoid and presphenoid bones to access the rostral and lateral aspects of the nasal conchae, turbinates and paranasal sinuses. The other cat skull was cut in a sagittal plane and the nasal and the frontal septum were sectioned to observe the medial aspect of these anatomical structures. 

#### 2.2.3. Macroscopic Sections

Five cat cadavers from the Necropsy Room were used to obtain anatomical sections. One cadaver was injected with blue latex through the external jugular veins, and another was injected with red and blue latex through the common carotid artery and the external jugular veins, respectively. These two specimens along with three other untreated cadavers were fixed by immersion in embalming solution for 7 days under refrigeration. Finally, the heads were sectioned with a band saw in transverse, sagittal and dorsal planes (0.5–0.7 cm wide).

### 2.3. Image Diagnostic Techniques (CT)

#### 2.3.1. Feline Skulls CT

Skulls from a lion, a cheetah and a cat were scanned in ventral recumbency using a dual-slice CT scanner (General Electric HiSpeed, General Electric Healthcare, Madrid, Spain) at the Veterinary Hospital of the University of Murcia. Technical parameters were 120 kV tube voltage, 80 mA tube current, 1 s tube rotation time, helical scan mode, collimator pitch of 1, 2–3 mm slice thickness and reconstructions interval with 50% overlap. Matrix dimensions were 512 × 512. Images were reviewed in a PACS workstation in bone window (WW = 1500, WL = 300).

#### 2.3.2. Feline Head CT

A head from each of these species (leopard, lion, cheetah and domestic cat) were positioned in ventral recumbency for tomographic evaluation by a 16-row scanner (Toshiba Asteliom, Toshiba Medical System, Madrid, Spain) at Veterinary Center of Levante Image Diagnostic, Ciudad Quesada, Rojales, Alicante, Spain. We used the following technical parameters for the four species: kVp 120, acquisition matrix 512 × 512, spiral pitch factor 0.625, and slice thickness 1.25 mm. A different mA was used depending on the species, ranging from 35 to 150 mA. The transverse original data were stored and transferred to the CT workstation. All images were analyzed using the DICOM viewer OsiriX MD 13.0.2 (Pixmeo, Bernex, Switzerland). Later, the DICOM images were postprocessed to obtain 3D MIP and surface and deep VR reconstructed images of these felids’ heads.

### 2.4. 3D Printing Models

A head each from a leopard, a lion, a cheetah and a domestic cat were used to obtain DICOM images. These images were loaded into AMIRA 5.6 software (Thermo Fisher Scientific, Waltham, MA, USA), where segmentation tools were used to delineate regions of interest (ROIs) in each set of CT images. Semi-automatic segmentation techniques such as thresholding were applied, and manual refinements were performed when necessary. From the segmented regions, 3D models were generated using AMIRA’s 3D reconstruction function, which were exported in STL (Standard Triangle Language) format. This format can represent the geometry of the surface of an object using a mesh of triangles, allowing its subsequent 3D printing. To prepare the STL images, the 3D Slicer software 3.0 was used to correct disjointed areas of the 3D models and join them to prevent them from falling out of the printed image. Subsequently, the prepared STL images were opened with the GrapbCad Print 1.71.7.21930 program (Waltham, MA, USA) and the parts to be printed were placed to scale. The print order was then sent to the Stratasys F170-FDM 3D printer (Los Angeles, CA, USA), which used an FDM (fused deposition modeling) system for the bony nasal cavity prints of the leopard, lion, cheetah and cat. Two types of materials were used for the prints: one for the support material and another for the model. For the support, a soluble material was used whose composition was largely polyvinyl alcohol. Acrylonitrile styrene acrylate was used for the model. Finally, the material was placed in a washing machine or centrifuge with sodium hydroxide for a period of 12 to 24 h, depending on the complexity of the preparation, which dissolved the support material. Finally, the models’ materials were isolated and then photographed.

## 3. Results

### 3.1. Anatomical Dissection

In the head dissection, it was possible to very precisely describe and identify the bony base of the nasal cavity, especially the nasal conchae, turbinates and paranasal sinuses. This dissection was performed in a domestic cat, taking this species as the type species. 

**Figure 1:**
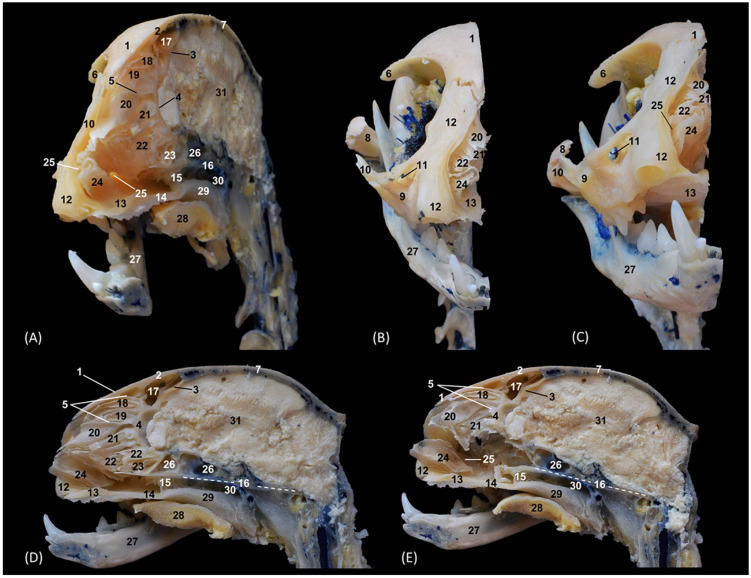
Representative anatomical dissections images of the cat’s head. The head was sectioned sagittally. The nasal and incisive bones were removed. (**A**): Left rostromedial view. (**B**): Rostrodorsal view. (**C**): Rostroventral view. (**D**): Left medial view with endoturbinated and ventral nasal concha. (**E**): Left medial view in which it has been removed, completely, the 2nd, 3rd and 4th endoturbinates. 1. Frontal bone; 2. Frontal bone: external plate; 3. Frontal bone: internal plate; 4. Ethmoid bone: cribriform plate; 5. Ethmoid bone: tectorial plate; 6. Frontal bone: zygomatic process; 7. Parietal bone; 8. Temporal bone: zygomatic process; 9. Zygomatic bone: body; 10. Zygomatic bone: temporal process; 11. Infraorbital canal; 12. Maxilla; 13. Maxilla: palatine process; 14. Palatine bone; 15. Choana; 16. Presphenoid, basisphenoid and basilar part of occipital bones; 17. Frontal sinus; 18. 1st ectoturbinate; 19. 2nd ectoturbinate; 20. Dorsal nasal concha; 21. Middle nasal concha; 22. 3rd endoturbinate; 23. 4th endoturbinate; 24. Ventral nasal concha; 25. Maxillary recess; 26. Sphenoidal sinus; 27. Right mandible; 28. Tongue; 29. Soft palate; 30. Nasopharynx (pars nasalis pharyngis); 31. Encephalon.

In Figure 1, we can observe from rostral and medial views the lateral wall and the structures that make up the interior of the nasal cavity. The nasal bones have been removed to better observe the nasal conchae in a rostral view. Therefore, we see in Figure 1 the squamous part of the frontal bone (external plate) that dorsally limits the nasal cavity and forms the roof of the frontal sinus. Furthermore, this sinus is closed caudally by the internal plate of the frontal bone. This plate continues ventrorostrally with the ethmoid bone (cribriform and tectorial plates), which closes the nasal cavity on its caudal side until it connects ventrally with the body of the presphenoid bone. The ventral limit of the nasal cavity is represented by the vomer, the palatine bone in the most caudal part and the palatine process of the maxilla. The most rostral part of the nasal cavity’s roof is established by the palatine process of the maxillary and the incisive bones. Laterally, the nasal cavity is limited by the maxilla. The rostral opening of the nasal cavity is bounded ventrally by the body and laterally by the nasal process of the incisive bone and dorsally by the rostral border of the nasal bone. The frontal sinus, undivided in the cat, contains ectoturbinates. In the preparation on its medial side we can observe, in this case, the first and second ectoturbinates. These ectoturbinates are supported by the tectorial plate of the ethmoid bone. The first of the nasal conchae to develop dorsally is the dorsal nasal concha and it does so from the cribriform plate of the ethmoid bone. It begins as a thin concha, but in the center, it widens before becoming very narrow in its most rostral part. The middle nasal concha has a more constant width along its entire length. The third endoturbinate is the most developed. It originates from two regions in the ethmoid bone; due to its great extension, it embraces and almost completely covers the ventral nasal concha on its medial side. The ventral nasal concha is observed after dissecting part of the third endoturbinate; it is topographically in the rostral half of the nasal cavity. Lateral to this turbinate is the narrow maxillary recess. The fourth endoturbinate is poorly developed and starts and projects from the opening of the sphenoidal sinus. Ventral and caudal to the endoturbinates, the choana or nasopharyngeal opening was observed.

**Figure 2:**
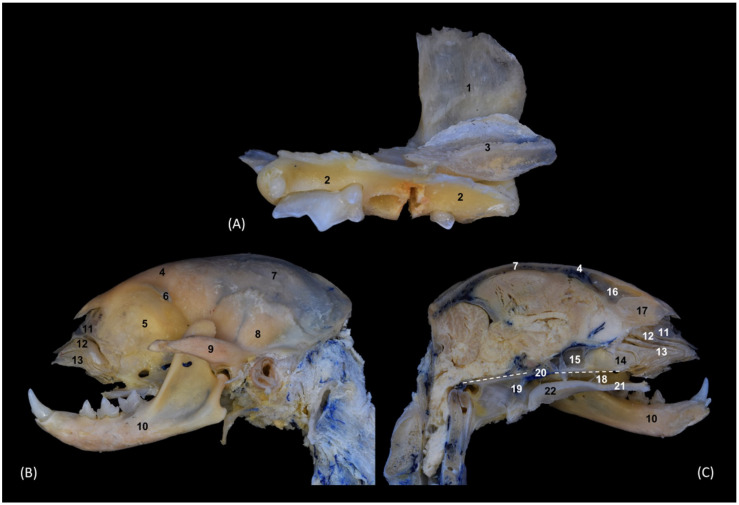
Representative anatomical dissections images of the cat’s head. (**A**): Medial view of the maxilla isolated. (**B**,**C**): Left lateral and right medial views, after removing the nasal, incisive, maxillary, zygomatic and lacrimal bones. 1. Maxilla: body; 2. Maxilla: palatine process; 3. Ventral nasal concha; 4. Frontal bone: external plate; 5. Frontal bone: orbital surface; 6. Frontal bone: zygomatic process; 7. Parietal bone; 8. Temporal bone: squamous part; 9. Temporal bone: zygomatic process; 10. Left mandible; 11. Dorsal nasal concha; 12. Middle nasal concha; 13. 3rd endoturbinate; 14. 4th endoturbinate; 15. Sphenoidal sinus; 16. Frontal sinus; 17. Nasal septum: cartilage (partially sectioned); 18. Choanae; 19. Nasopharynx (pars nasalis pharyngis); 20. Presphenoid, basisphenoid and basilar part of occipital bones; 21. Palatine bone. 22. Soft palate.

In Figure 2, the maxilla was dissected to observe that the ventral nasal concha, which is feather-shaped, is short and is only attached to the maxilla. After resecting the maxilla, it can be observed how the dorsal and middle conchae and the third and fourth endoturbinates are connected to the ethmoid bone and are independent of the maxilla.

### 3.2. Trepanations

**Figure 3:**
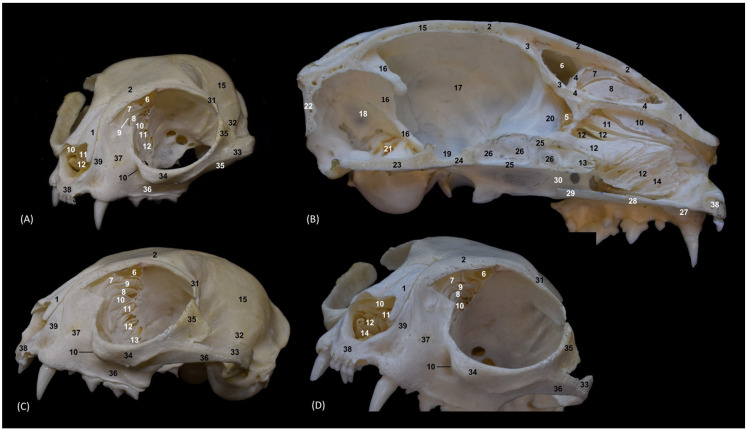
Bone trepanations images of the cat’s skull. (**A**): Left rostrolateral view after removing the orbital surface of the frontal bone and the wings of the presphenoid and basisphenoid bones. (**B**): Right medial view after removing the bone and cartilaginous parts of the nasal septum. (**C**): Left dorsal oblique view after removing the rostral half of the orbit. (**D**): Left dorsal oblique view showing the endoturbinates and ectoturbinates. 1. Nasal bone; 2. Frontal bone: external plate; 3. Frontal bone: internal plate; 4. Ethmoid bone: tectorial plate; 5. Ethmoid bone: cribriform plate; 6. Frontal sinus; 7. 1st ectoturbinate; 8. 2nd ectoturbinate; 9. 3rd ectoturbinate; 10. Dorsal nasal concha; 11. Middle nasal concha; 12. 3rd endoturbinate; 13. 4th endoturbinate; 14. Ventral nasal concha; 15. Parietal bone; 16. Osseous tentorium of the cerebellum; 17. Cerebral fossa; 18. Cerebellar fossa; 19. Hypophyseal fossa; 20. Ethmoidal fossa; 21. Temporal bone: petrous part; 22. Occipital bone: squama; 23. Occipital bone: basilar part; 24. Basisphenoid bone: body; 25. Presphenoid bone: body; 26. Sphenoidal sinus: septum; 27. Incisive bone: palatine process; 28. Maxilla: palatine process; 29. Palatine bone: horizontal and perpendicular plate; 30. Choana; 31. Frontal bone: zygomatic process; 32. Temporal bone: squamous part; 33. Temporal bone: zygomatic process; 34. Zygomatic bone: body; 35. Zygomatic bone: frontal process; 36. Zygomatic bone: temporal process; 37. Maxilla; 38. Incisive bone: body; 39: Incisive bone: nasal process.

The two domestic cat skulls with trepanations presented in this study allowed us to identify the entire origin and route of the ethmoturbinates, as well as the bones that support the bony nasal cavity. After cutting part of the orbital surface of the frontal bone, and the wings of the basisphenoid and presphenoid bones, the origin of the three ectoturbinates can be visualized from the tectorial plate of the ethmoid bone. The first and the second ectoturbinates reach great development on their medial side, while the third is sometimes camouflaged between the other two, since it develops more laterally. The three ectoturbinates were delimited dorsally and ventrally by the tectorial plate of the ethmoid bone (Figure 3B). In this same image, below the dorsal and middle nasal conchae, the great development of the third endoturbinate and its trabeculated bone appearance can be seen. This endoturbinate covers the dorsal half of the medial face of the ventral nasal concha and its double origin in the ethmoid bone can be seen again on both its lateral (Figure 3C) and its medial faces (Figure 3B). The fourth endoturbinate, small in size, was located next to the sphenoid sinus. In the oblique views (Figure 3A,C,D), it was observed how these conchae reach the nasal vestibule, being very thin and fragile bony nasal conchae.

### 3.3. Sectional Anatomy

The anatomy of the bony nasal cavity was described through transverse, dorsal and sagittal sections of the domestic cat heads (Figure 4, Figure 5, Figure 6, Figure 7, Figure 8, Figure 9, Figure 10, Figure 11, Figure 12, Figure 13, Figure 14, Figure 15, Figure 16, Figure 17 and Figure 18).

**Figure 4:**
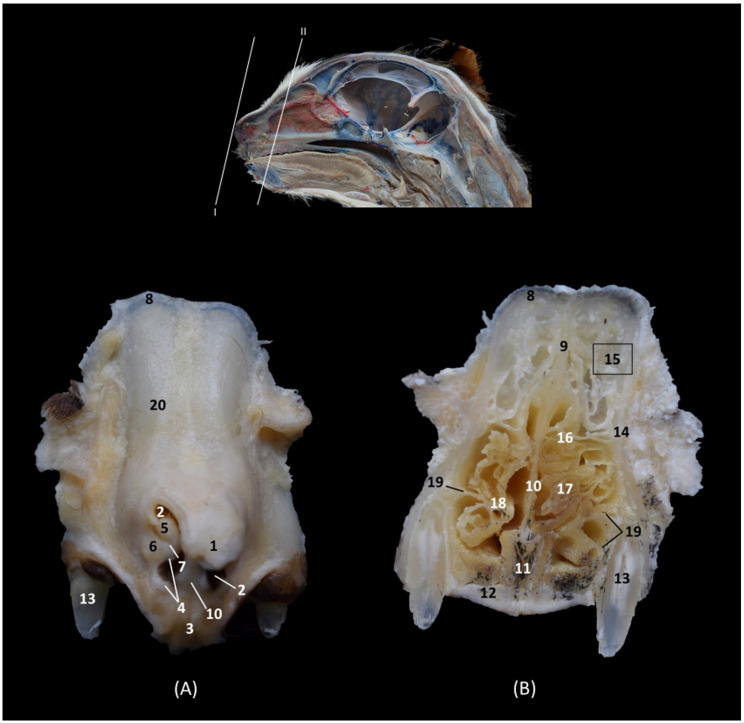
Transverse anatomical sections of the cat’s head at the level of the nasal plane, vestibule and rostral part of the nasal cavity: respiratory part. (**A**): Rostral view, level section I. (**B**): Caudal view, level section II. 1. Tip of the nose; 2. Nasal orifice (nares); 3. Subnasal groove or philtrum; 4. Lateral accessory nasal cartilage; 5. Dorsal lateral nasal cartilage; 6. Ventral lateral nasal cartilage; 7. Alar groove; 8. Frontal bone; 9. Frontal sinus: septum; 10. Nasal septum: cartilage; 11. Vomer; 12. Maxilla: palatine process; 13. Canine tooth: root; 14. Maxilla: orbital surface; 15. Ectoturbinates; 16. Dorsal and middle nasal conchae; 17. 3rd endoturbinate; 18. Ventral nasal concha; 19. Conchal crest of the maxilla; 20. Nasal bone.

**Figure 5:**
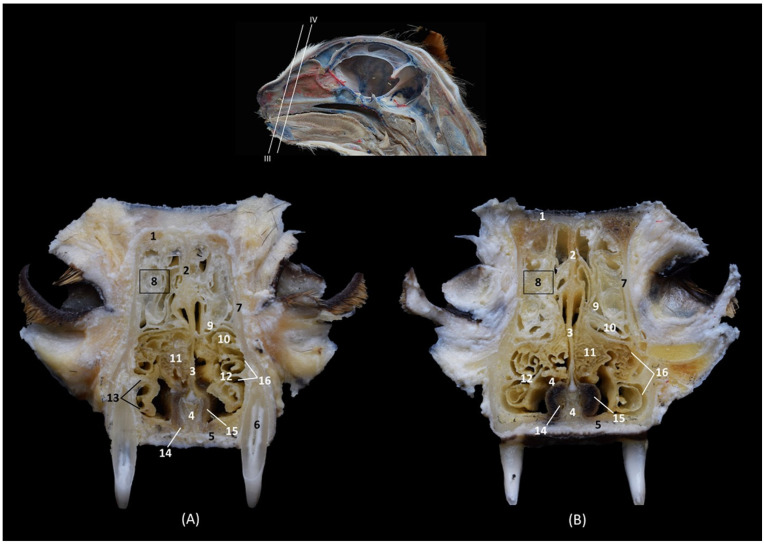
Transverse anatomical sections of the cat’s head at the level of the rostral part of the nasal cavity: respiratory part. (**A**) Rostral view, level section III. (**B**) Caudal view, level section IV. 1. Frontal bone; 2. Frontal sinus: septum; 3. Nasal septum: cartilage; 4. Vomer; 5. Maxilla: palatine process; 6. Canine tooth: root; 7. Maxilla: orbital surface; 8. Ectoturbinates; 9. Dorsal nasal concha; 10. Middle nasal concha; 11. 3rd endoturbinate; 12. Ventral nasal concha; 13. Conchal crest of the maxilla; 14. Vomeronasal organ; 15. Nasal cavernous plexuses; 16. Maxillary recess.

**Figure 6:**
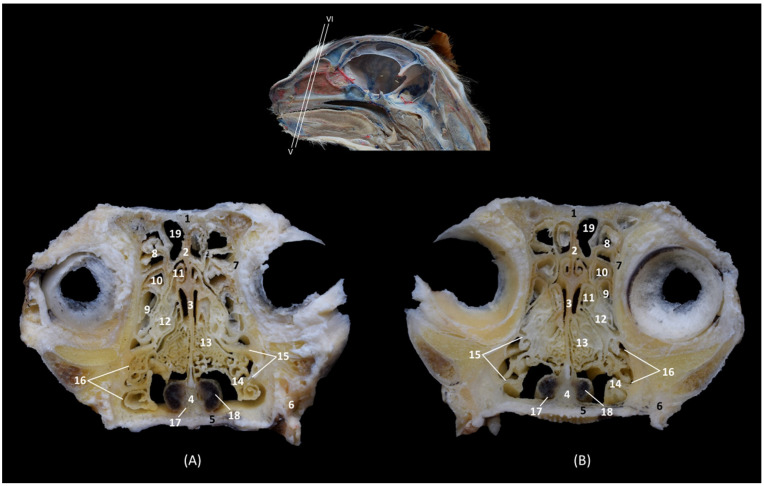
Transverse anatomical sections of the cat’s head at the level of the middle part of the nasal cavity; respiratory part. (**A**): Rostral view, level section V. (**B**): Caudal view, level section VI. 1. Frontal bone; 2. Frontal sinus: septum; 3. Nasal septum: cartilage; 4. Vomer; 5. Maxilla: palatine process; 6. 1st premolar tooth: root; 7. Frontal bone: orbital surface; 8. 1st ectoturbinate; 9. 2nd ectoturbinate; 10. 3rd ectoturbinate; 11. Dorsal nasal concha; 12. Middle nasal concha; 13. 3rd endoturbinate; 14. Ventral nasal concha; 15. Conchal crest of the maxilla; 16. Maxillary recess; 17. Vomeronasal organ; 18. Nasal cavernous plexuses; 19. Frontal sinus.

**Figure 7:**
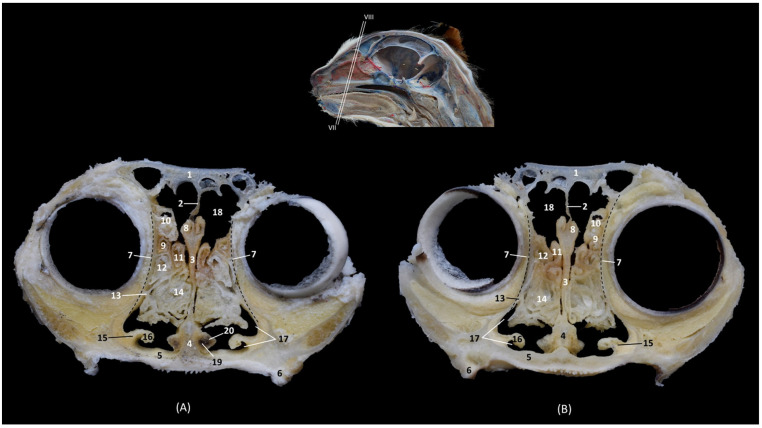
Transverse anatomical sections of the cat’s head at the level of the caudal part of the nasal cavity; respiratory part. (**A**): Rostral view, level section VII. (**B**): Caudal view, level section VIII. 1. Frontal bone; 2. Frontal sinus: septum; 3. Nasal septum: cartilage; 4. Vomer; 5. Maxilla: palatine process; 6. 2nd premolar tooth: root; 7. Frontal bone: orbital surface; 8. 1st ectoturbinate; 9. 2nd ectoturbinate; 10. 3rd ectoturbinate; 11. Dorsal nasal concha; 12. Middle nasal concha; 13. Conchal crest of the 3rd endoturbinate; 14. 3rd endoturbinate; 15. Conchal crest of the maxilla; 16. Ventral nasal concha; 17. Maxillary recess; 18. Frontal sinus; 19. Vomeronasal organ; 20. Nasal cavernous plexuses.

**Figure 8:**
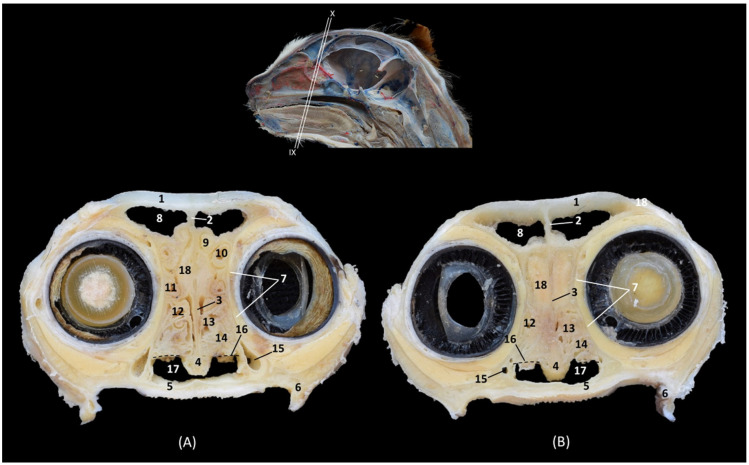
Transverse anatomical sections of the cat’s head at the level of the caudal part of the nasal cavity; olfactory part. (**A**): Rostral view, level section IX. (**B**): Caudal view, level section X. 1. Frontal bone; 2. Frontal sinus: septum; 3. Ethmoid bone: perpendicular plate; 4. Vomer; 5. Maxilla: palatine process; 6. 3rd premolar tooth: root; 7. Ethmoid bone: orbital plate; 8. Frontal sinus; 9. 1st ectoturbinate; 10. 2nd ectoturbinate; 11. Dorsal nasal concha; 12. Middle nasal concha; 13. 3rd endoturbinate; 14. 4th endoturbinate; 15. Maxillary recess; 16. Ethmoid bone: basal plate; 17. Choana; 18. Olfactory bulb.

**Figure 9:**
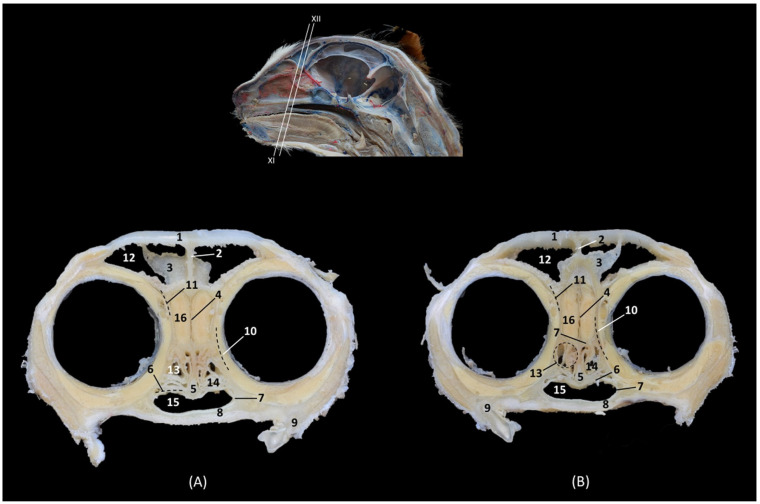
Transverse anatomical sections of the cat’s head at the level of the caudal part of the nasal cavity: olfactory part. (**A**) Rostral view, level section XI. (**B**) Caudal view, level section XII. 1. Frontal bone; 2. Frontal sinus: septum; 3. Ethmoid bone: tectorial plate; 4. Ethmoid bone: perpendicular plate; 5. Vomer; 6. Ethmoid bone: basal plate; 7. Palatine bone: perpendicular plate; 8. Palatine bone: horizontal plate; 9. Molar tooth: root; 10. Presphenoid wing: orbital surface; 11. Frontal bone: orbital surface; 12. Frontal sinus; 13. 3rd endoturbinate; 14. 4th endoturbinate; 15. Choana; 16. Olfactory bulb.

**Figure 10:**
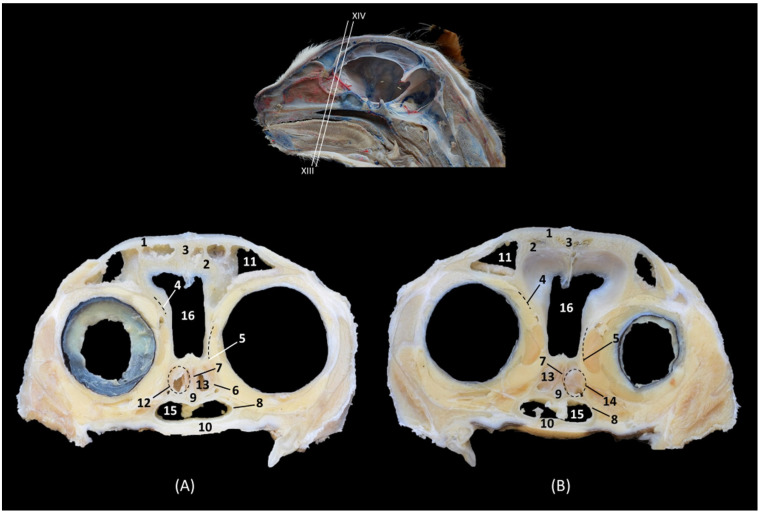
Transverse anatomical sections of the cat’s head at the level of the paranasal sinuses and nasopharynx (pars nasalis pharyngis). (**A**): Rostral view, level section XIII. (**B**): Caudal view, level section XIV. 1. Frontal bone: external plate; 2. Frontal bone: internal plate; 3. Frontal sinus: septum; 4. Frontal bone: orbital surface; 5. Basisphenoid bone: wing (orbital surface); 6. Presphenoid bone: body; 7. Sphenoidal sinus: septum; 8. Basisphenoid bone: pterygoid process; 9. Vomer; 10. Soft palate; 11. Frontal sinus; 12. Sphenoidal sinus; 13. 4th endoturbinate; 14. Sphenoidal sinus: caudal wall; 15. Nasopharynx (pars nasalis pharyngis); 16. Cranial cavity.

**Figure 11:**
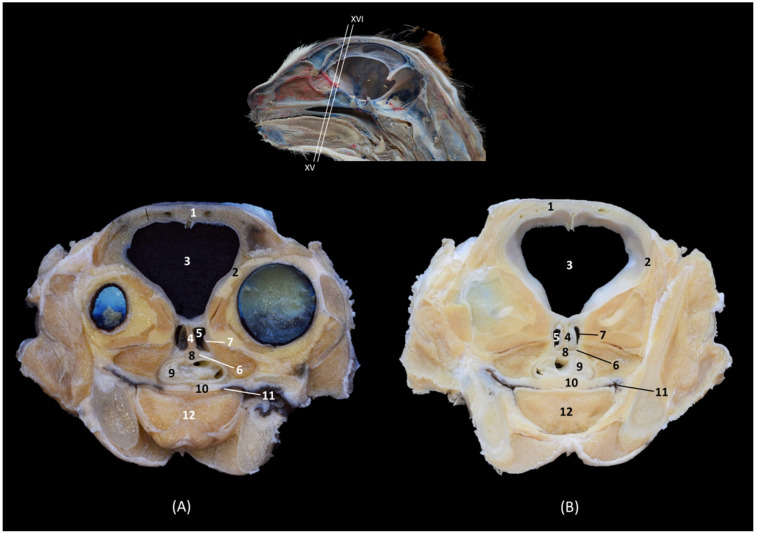
Transverse anatomical sections of the cat’s head at the level of the paranasal sinuses and nasopharynx (pars nasalis pharyngis). (**A**): Rostral view, level section XV. (**B**): Caudal view, level section XVI. 1. Frontal bone; 2. Frontal bone: orbital surface; 3. Cranial cavity; 4. Sphenoidal sinus: septum; 5. Sphenoidal sinus; 6. Presphenoid bone: body; 7. Presphenoid bone: wing; 8. Vomer; 9. Nasopharynx (pars nasalis pharyngis): mucosa; 10. Soft palate; 11. Oropharynx; 12. Tongue.

In the most rostral view of the transverse sections (Figure 4, Figure 5, Figure 6, Figure 7, Figure 8, Figure 9, Figure 10 and Figure 11), the nasal bone can be observed covering the most rostral part of the nasal cavity (Figure 4A). In this figure, we can also see the cartilages that make up the external nose and maintain and support the nostrils. Thus, we can observe the dorsal lateral cartilage of the nose forming the anatomical base of the dorsum of the openings. The ventral lateral cartilage limits the ventrolateral part of the nose, and the alar fold closes it laterally. The alar groove was described in the ventrolateral area of the opening. Once inside the nasal cavity and in the caudal cross-sectional view (Figure 4B), the framework of the ectoturbinates was observed in the frontal sinus. The dorsal and middle nasal conchae were seen, and the third endoturbinate can also be observed in a more medial position. The ventral nasal concha could be seen sectioned with one or two conchal crests of the maxilla depending on the section’s symmetry.

In the next section (Figure 5), the septum of the frontal sinus is already observed. The dorsal and middle nasal conchae appeared quite narrow and displaced at this level, and the third endoturbinate begins to increase in size. The ventral nasal concha was observed at this level in its maximum development, and the maxillary recess develops on its lateral surface. At this level, the nasal cavernous plexuses reach great development and the vomeronasal organ was well appreciated.

In the following transverse section (Figure 6), in the medial part of the nasal cavity, the ventral nasal concha was already decreasing in size while the third endoturbinate reaches its greatest development. At this level, the dorsal nasal concha also increases in size. Furthermore, the three ectoturbinates could be clearly identified. From dorsal to ventral, the first, the third and the second ectoturbinates were observed.

In the next transverse section (Figure 7), the end of the ventral nasal concha was seen. At this level a lateral attachment of the third endoturbinate to the frontal bone could also be identified. Ectoturbinates also decrease at this level and the greater size of the frontal sinus was observed. At this stage, the vomeronasal organ reaches its caudal extent.

Figure 8 continues with a caudal section of the nasal cavity where the olfactory bulb was already sectioned. The nasal septum is replaced at this level by the perpendicular plate of the ethmoid bone and the third endoturbinate was no longer observed. The fourth endoturbinate begins to be appreciated and the choana or nasopharyngeal opening was already identified ventrally between the basal plate of the ethmoid bone and palatine process of the maxilla.

In Figure 9, the tectorial plate of the ethmoid bone can be visualized independently as a plate that embraces the ectoturbinates dorsally and ventrally. The fourth endoturbinate is larger in this section.

Figure 10 shows both the external and the internal plates of the frontal bone. At this level, we could also observe the fourth endoturbinate in the sphenoidal sinus, which has a medial septum that divides it into two.

In this last transverse section (Figure 11), we observed dorsal to the nasopharynx (pars nasalis pharyngis) the caudal extension of the sphenoidal sinus.

**Figure 12:**
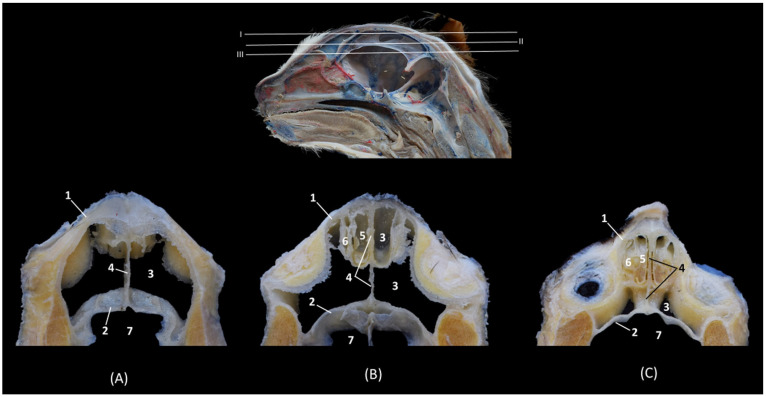
Dorsal anatomical sections of the cat’s head at the level of the (**A**) frontal sinus, level I and (**B**,**C**) frontal sinus and ectoturbinates, levels II and III. Images are oriented so that the rostral part is at the top. All views are dorsal. 1. Frontal bone: external plate; 2. Frontal bone: internal plate; 3. Frontal sinus; 4. Frontal sinus: septum; 5. 1st ectoturbinate; 6. 3rd ectoturbinate; 7. Cranial cavity.

**Figure 13:**
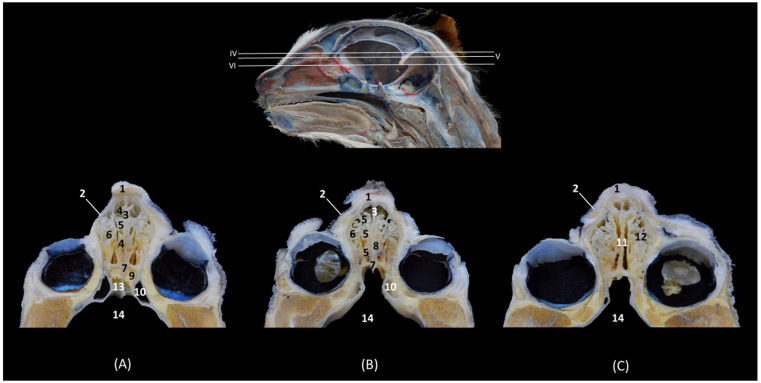
Dorsal anatomical sections of the cat’s head at the level of the (**A**,**B**) ectoturbinates, levels IV and V, and (**C**) endoturbinates, level VI. Images are oriented so that the rostral part is at the top. All views are dorsal. 1. Nasal bone; 2. Maxilla; 3. Frontal sinus: septum; 4. 1st ectoturbinate; 5. 2nd ectoturbinate; 6. 3rd ectoturbinate; 7. Frontal bone: internal plate; 8. Ethmoid bone: tectorial plate; 9. Ethmoid bone: cribriform plate; 10. Frontal sinus; 11. Nasal septum: cartilage; 12. Dorsal nasal concha; 13. Ethmoidal fossa; 14. Cranial cavity.

**Figure 14:**
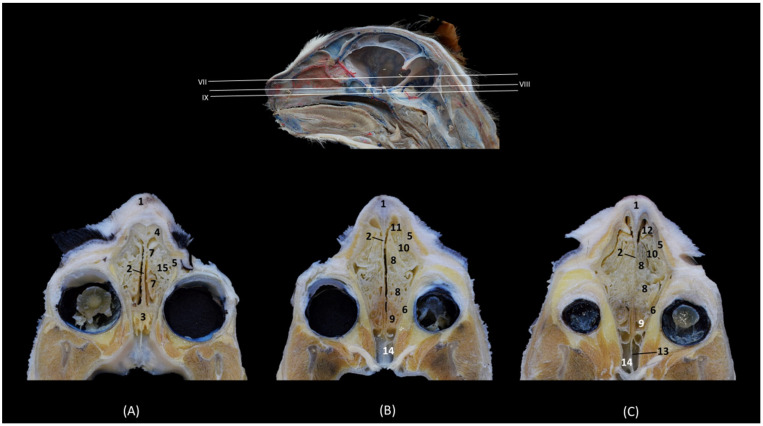
Dorsal anatomical sections at the level of cat’s head at the level of the (**A**,**B**) endoturbinates, levels VII and VIII, and (**C**) sphenoidal sinuses, level IX. Images are oriented so that the rostral part is at the top. All views are dorsal. 1. Nose; 2. Nasal septum: cartilage; 3. Ethmoid bone: cribriform plate; 4. Incisive bone: nasal process; 5. Maxilla; 6. Presphenoid bone; 7. Middle nasal concha; 8. 3rd endoturbinate; 9. 4th endoturbinate; 10. Ventral nasal concha; 11. Basal fold; 12. Alar fold; 13. Sphenoidal sinus: septum; 14. Sphenoidal sinus; 15. Dorsal nasal concha.

**Figure 15:**
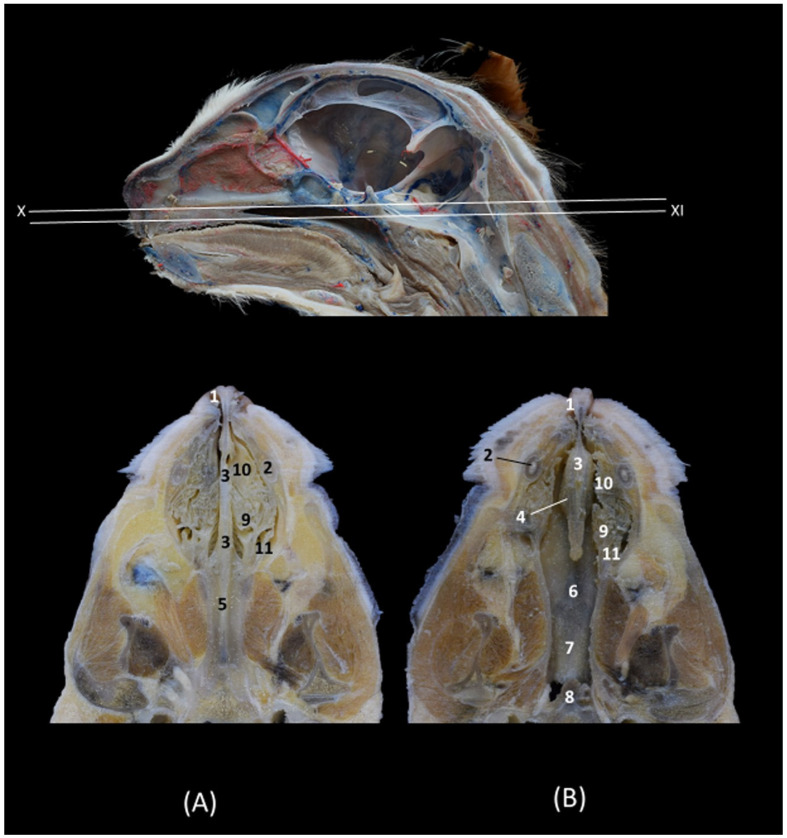
Dorsal anatomical sections of the cat’s head at the level of the (**A**) endoturbinates and ventral nasal concha, level X and (**B**) nasopharynx (pars nasalis pharyngis), level XI. Images are oriented so that the rostral part is at the top. All views are dorsal. 1. Nasal orifice; 2. Canine tooth: root; 3. Vomer; 4. Nasal cavernous plexuses; 5. Presphenoid bone; 6. Choana; 7: Soft palate: dorsal surface; 8. Nasopharynx (pars nasalis pharyngis); 9. 3rd endoturbinate; 10. Ventral nasal concha; 11. 4th endoturbinate.

The dorsal sections (Figure 12, Figure 13, Figure 14 and Figure 15) show, in the most dorsal section (Figure 12A), the external and internal plates of the frontal bone, outlining the frontal sinus and their septum. In the next sections (Figure 12B,C), the first ectoturbinate was located inside the frontal sinus, and lateral to it is the third ectoturbinate.

In the next section, more caudal in position (Figure 13A,B), the ectoturbinates could be visualized in their maximum development. The first ectoturbinate was within the most dorsal part of and attached to the septum of the frontal sinus. The second ectoturbinate could also be visualized, and the third ectoturbinate appeared to be more developed on the lateral face. In Figure 13C, the dorsal nasal concha begins to be observed.

Ventral to this, in Figure 14A,B, the middle nasal concha and the third endoturbinate can be seen, sections in which the entire extent of the latter can be clearly appreciated. The ventral nasal concha was observed in the most rostral area, lateral to the third endoturbinate. Additionally, in Figure 14C, the alar fold continues with the ventral nasal concha. In these sections, we could also see the sphenoidal sinus and the fourth endoturbinate.

In the most ventral dorsal section of the nasal cavity (Figure 15), the ventral nasal concha and the third and fourth endoturbinates were clearly observed. In addition to the palatine bone, the ventral part of the floor of the nasal cavity was represented by the vomer. The nasal cavernous plexuses were seen, but the vomeronasal organ was not observed. The space between the vomer and palatine bone and the soft palate forms the choana or nasopharyngeal opening.

**Figure 16:**
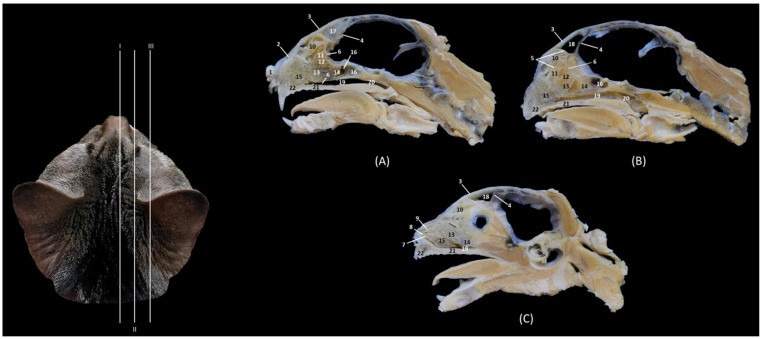
Sagittal anatomical sections of the cat’s head at the level of the (**A**,**B**) paranasal sinuses, nasal cavity and nasopharynx (pars nasalis pharyngis), levels I and II, and (**C**) frontal sinuses and nasal cavity, level III. Images are oriented so that the rostral part is to the left and the dorsal is at the top. All views are left lateral. 1. Nose; 2. Nasal bone; 3. Frontal bone: external plate; 4. Frontal bone: internal plate; 5. Ethmoid bone: tectorial plate; 6. Ethmoid bone: cribriform plate; 7. Basal fold; 8. Alar fold; 9. Straight fold; 10. Ectoturbinates; 11. Dorsal nasal concha; 12. Middle nasal concha; 13. 3rd endoturbinate; 14. 4th endoturbinate; 15. Ventral nasal concha; 16. Sphenoidal sinus; 17. Frontal sinus: septum; 18. Frontal sinus; 19. Choana; 20. Nasopharynx (pars nasalis pharyngis); 21. Maxilla: palatine process; 22. Incisive bone.

**Figure 17:**
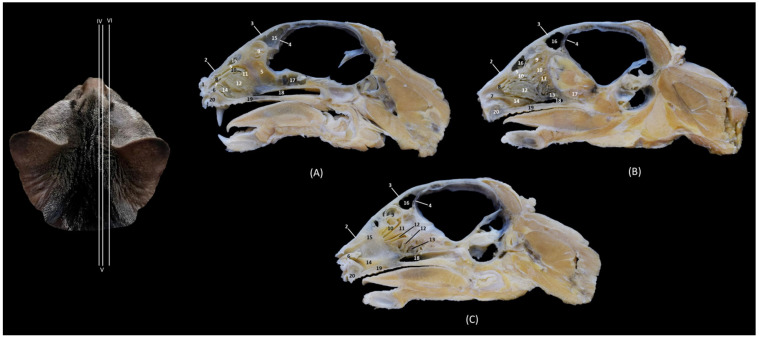
Sagittal anatomical sections of the cat’s head at the level of the (**A**–**C**) nasal cavity, paranasal sinuses and nasopharynx (pars nasalis pharyngis), levels IV, V and VI. Images are oriented so that the rostral part is to the left and the dorsal is at the top. All views are left lateral. 1. Nose; 2. Nasal bone; 3. Frontal bone: external plate; 4. Frontal bone: internal plate; 5. Ethmoid bone: tectorial plate; 6. Basal fold; 7. Alar fold; 8. Straight fold; 9. Ectoturbinates; 10. Dorsal nasal concha; 11. Middle nasal concha; 12. 3rd endoturbinate; 13. 4th endoturbinate; 14. Ventral nasal concha; 15. Frontal sinus: septum; 16. Frontal sinus; 17. Sphenoidal sinus; 18. Choana; 19. Maxilla: palatine process; 20. Incisive bone.

**Figure 18:**
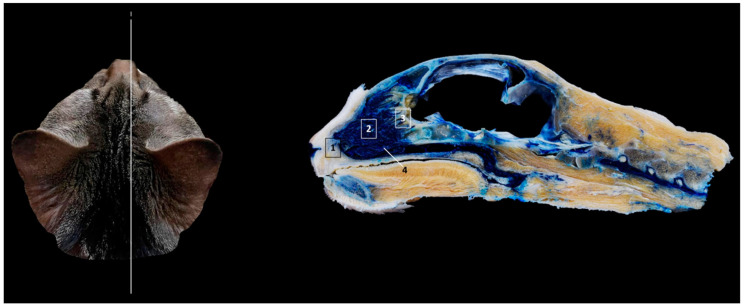
Sagittal anatomical section of the cat’s head at the level of the nasal cavity and paranasal sinuses, level I. The venous plexuses have been injected with blue latex via an external jugular vein. The image is oriented so that the rostral part is to the left and the dorsal is at the top. Left lateral view. 1. Nasal vestibule; 2. Respiratory part; 3. Olfactory part; 4. Nasal cavernous plexuses.

In the sagittal sections of the nasal cavity (Figure 16, Figure 17 and Figure 18), the ectoturbinates are observed in the frontal sinus, and the endoturbinates begin to extend from the cribriform plate of the ethmoid bone. In addition, the ventral nasal concha was seen to be continuous rostrally with the alar and basal folds, while the dorsal nasal concha was continuous with the straight fold. The bullas of the dorsal and ventral nasal conchae were not identified (Figure 16).

In the next anatomical sections shown in Figure 17, the entire longitudinal path of the conchae can be seen, along with the maximum extent of the third endoturbinate, while the ventral nasal concha was limited only to the cranial half of the nasal cavity (Figure 17A). In the more sagittal sections (Figure 17B,C), the fourth endoturbinate close to the sphenoidal sinus could already be identified, but it was not observed in the sagittal section.

Finally, Figure 18 shows a sagittal section after injection of blue latex to better highlight the vascularization of the respiratory part of the nasal cavity. It is worth noting the extensive blood supply to the respiratory part of the nasal cavity due to the large number of nasal cavernous plexuses that exist at this level and their scarcity in the olfactory part.

### 3.4. Felid Skulls CT

**Figure 19:**
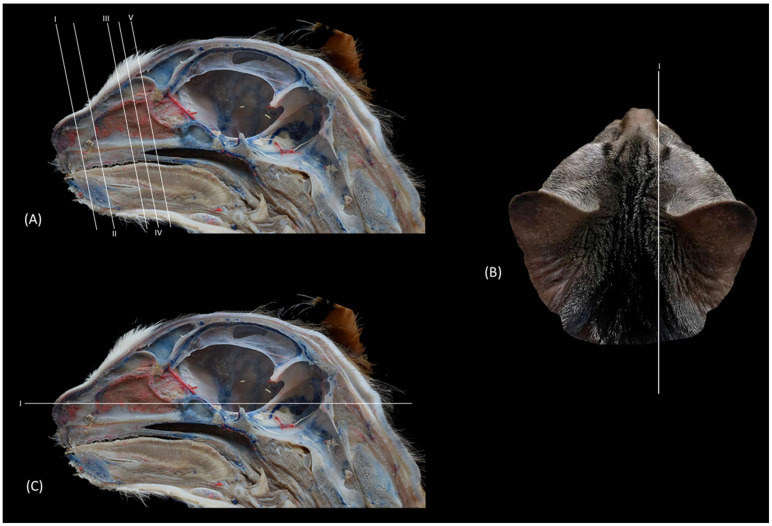
Approximate anatomical-level CT sections of the cat’s head. Lines depict the transverse (**A**), sagittal (**B**) and dorsal (**C**) planes. Each number represents the location for each CT transverse (I to V), sagittal (I) and dorsal (I) images.

**Figure 20:**
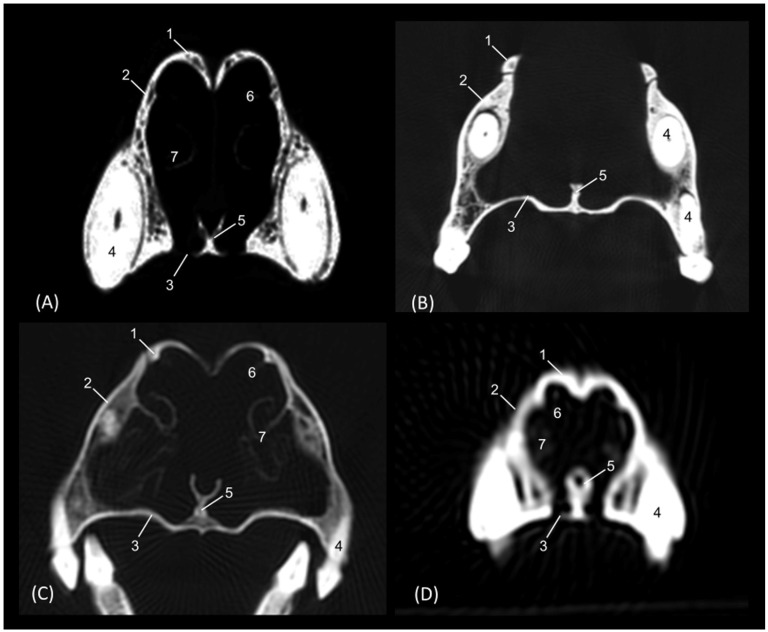
Amira representative transverse CT images at level of the rostral portion of the respiratory part, level I. Images are oriented so that the left side of the head is to the right and the dorsal is at the top. All views are rostral. (**A**): Leopard; (**B**): Lion; (**C**): Cheetah and (**D**): Cat. 1. Nasal bone; 2. Maxilla; 3. Maxilla: palatine process; 4. Canine tooth: root; 5. Vomer; 6. Dorsal nasal concha; 7. Ventral nasal concha.

**Figure 21:**
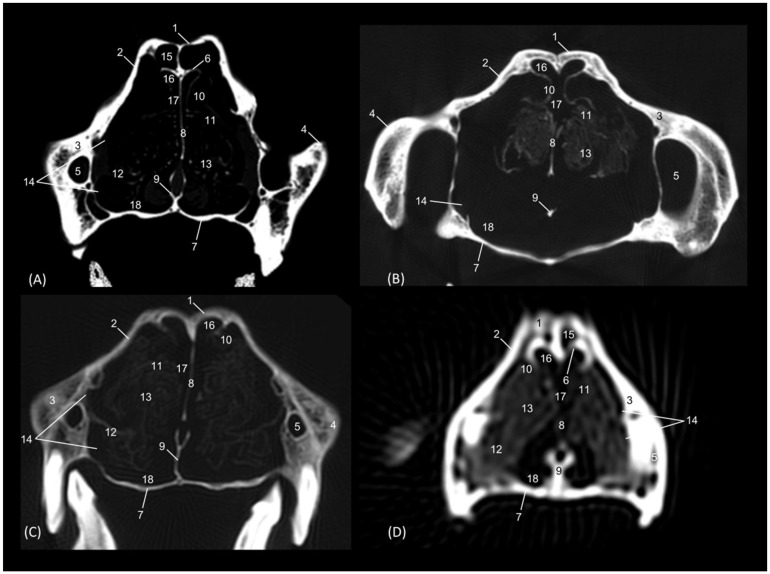
Amira representative transverse CT images at level of the middle portion of the respiratory part, level II. Images are oriented so that the left side of the head is to the right and the dorsal is at the top. All views are rostral. (**A**): Leopard; (**B**): Lion; (**C**): Cheetah and (**D**): Cat. 1. Nasal bone; 2. Frontal bone; 3. Maxilla; 4. Zygomatic bone; 5. Infraorbital canal; 6. Ethmoid bone: tectorial plate; 7. Maxilla: palatine process; 8. Nasal septum; 9. Vomer; 10. Dorsal nasal concha; 11. Middle nasal concha; 12. Ventral nasal concha; 13. 3rd endoturbinate; 14. Maxillary recess; 15. Frontal sinus; 16. Dorsal nasal meatus; 17. Common nasal meatus; 18. Ventral nasal meatus.

**Figure 22:**
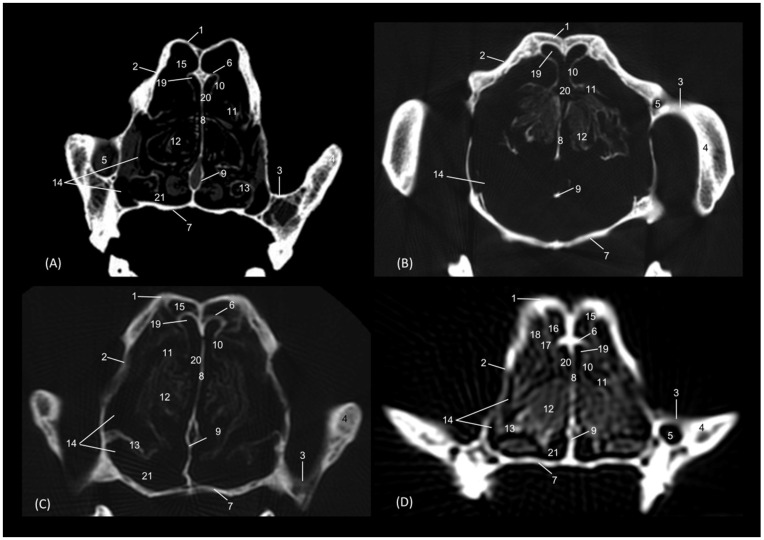
Amira representative transverse CT images at level of the middle portion of the respiratory part, level III. Images are oriented so that the left side of the head is to the right and the dorsal is at the top. All views are rostral. (**A**): Leopard; (**B**): Lion; (**C**): Cheetah and (**D**): Cat. 1. Frontal bone; 2. Maxilla; 3. Zygomatic process: body; 4. Zygomatic bone: temporal process; 5. Infraorbital canal; 6. Ethmoid bone: tectorial plate; 7. Maxilla: palatine process; 8. Nasal septum; 9. Vomer; 10. Dorsal nasal concha; 11. Middle nasal concha; 12. 3rd endoturbinate; 13. Ventral nasal concha; 14. Maxillary recess; 15. Frontal sinus; 16. 1st ectoturbinate; 17. 2nd ectoturbinate; 18. 3rd ectoturbinate; 19. Dorsal nasal meatus; 20. Common nasal meatus; 21. Ventral nasal meatus.

**Figure 23:**
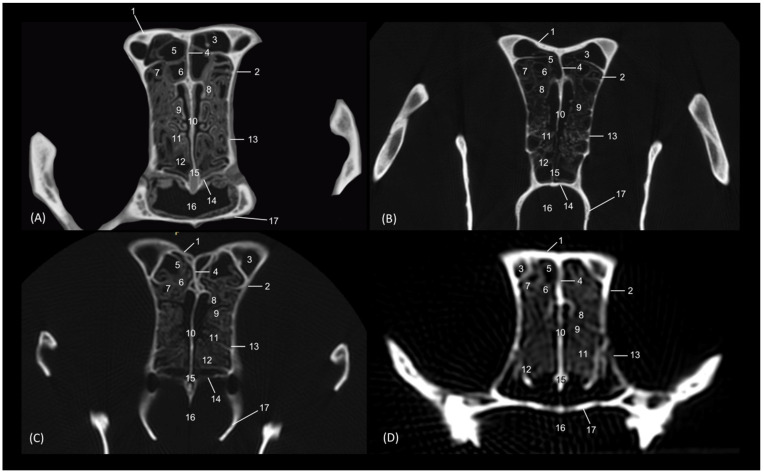
Amira representative transverse CT images at the level of the caudal portion of the respiratory part, level IV. Images are oriented so that the left side of the head is to the right and the dorsal is at the top. All views are rostral. (**A**): Leopard; (**B**): Lion; (**C**): Cheetah and (**D**): Cat. 1. Frontal bone: external plate; 2. Frontal bone: orbital surface; 3. Frontal sinus; 4. Frontal sinus: septum; 5. 1st ectoturbinate; 6. 2nd ectoturbinate; 7. 3rd ectoturbinate; 8. Dorsal nasal concha; 9. Middle nasal concha; 10; Ethmoid bone: perpendicular plate; 11. 3rd endoturbinate; 12. 4th endoturbinate; 13. Presphenoid bone: wing (orbital surface); 14. Presphenoid bone: body; 15. Vomer; 16. Choana; 17. Palatine bone: horizontal and perpendicular plates.

**Figure 24:**
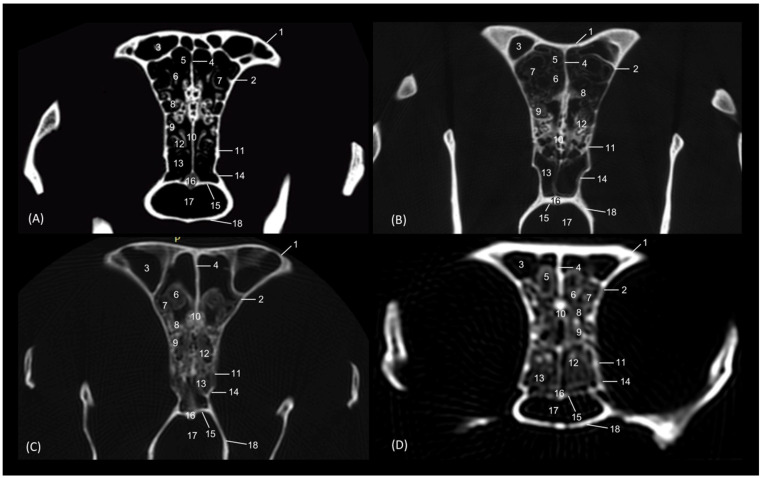
Amira representative transverse CT images at the level of the olfactory part of the nasal cavity, level V. Images are oriented so that the left side of the head is to de right and the dorsal is at the top. All views are rostral. (**A**): Leopard; (**B**): Lion; (**C**): Cheetah and (**D**): Cat. 1. Frontal bone: external plate; 2. Frontal bone: orbital surface; 3. Frontal sinus; 4. Frontal sinus: septum; 5. 1st ectoturbinate; 6. 2nd ectoturbinate; 7. 3rd ectoturbinate; 8. Dorsal nasal concha; 9. Middle nasal concha; 10. Ethmoid bone: perpendicular plate; 11. Basisphenoid bone: wing (orbital surface); 12. 3rd endoturbinate; 13. 4th endoturbinate; 14. Presphenoid bone: wing; 15. Presphenoid bone: body; 16. Vomer; 17. Choana; 18. Palatine bone: horizontal and perpendicular plates.

**Figure 25:**
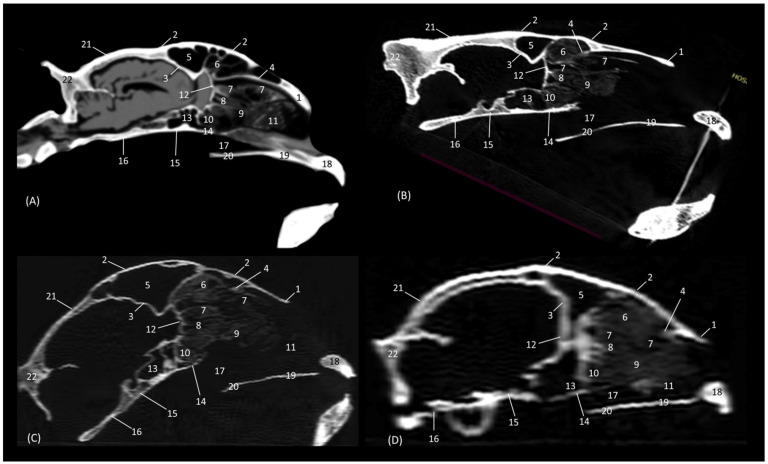
Amira representative sagittal multiplanar reconstruction (MPR) CT images at the the level of the nasal cavity and paranasal sinuses, level I. Images are oriented so that the rostral part is to the right and dorsal is at the top. All views are right lateral. (**A**): Leopard; (**B**): Lion; (**C**): Cheetah and (**D**): Cat. 1. Nasal bone; 2. Frontal bone: external plate; 3. Frontal bone: internal plate; 4. Ethmoid bone: tectorial plate; 5. Frontal sinus; 6. Ectoturbinates (1st, 2nd and 3rd); 7. Dorsal nasal concha; 8. Middle nasal concha; 9. 3rd endoturbinate; 10. 4th endoturbinate; 11. Ventral nasal concha; 12. Ethmoid bone: cribriform plate; 13. Sphenoidal sinus; 14. Presphenoid bone: body; 15. Basisphenoid bone: body; 16. Occipital bone: basilar part; 17. Choana; 18. Incisive bone: palatine process; 19. Maxilla: palatine process; 20. Palatine bone: horizontal plate; 21. Parietal bone; 22. Occipital bone: squama.

**Figure 26:**
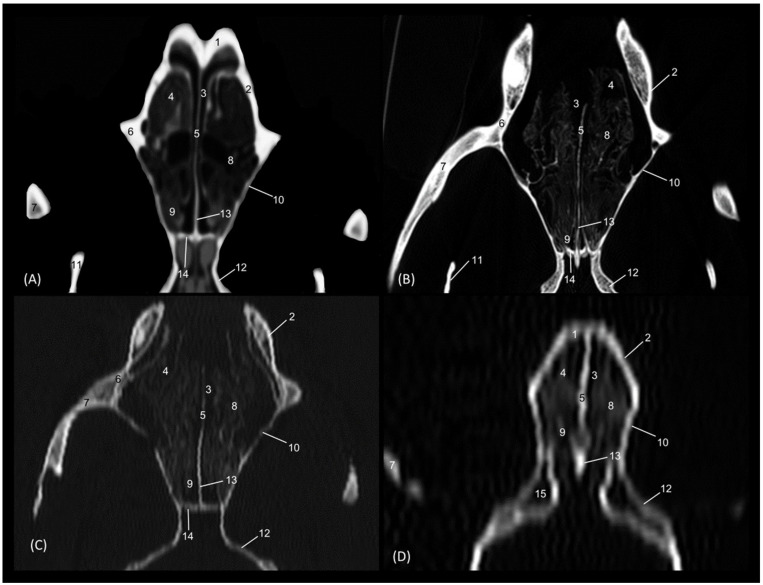
Amira representative dorsal multiplanar reconstruction (MPR) CT images at level of the 3rd and 4th endoturbinates, level I. Images are oriented so that the rostral part is at the top. All views are dorsal. (**A**): Leopard; (**B**): Lion; (**C**): Cheetah and (**D**): Cat. 1. Incisive bone; 2. Maxilla; 3. Common nasal meatus; 4. Ventral nasal concha; 5. Nasal septum; 6. Zygomatic bone: body; 7. Zygomatic bone: temporal process; 8. 3rd endoturbinate; 9. 4th endoturbinate; 10. Frontal bone: orbital surface; 11. Mandible: ramus; 12. Presphenoid bone: wing; 13. Ethmoid bone: perpendicular plate; 14. Ethmoid bone: cribriform plate; 15. Frontal sinus.

The section levels of each plane are represented in Figure 19. The CT study of the felid skull allowed for the identification in the three spatial anatomical planes of the bony base that makes up this cavity: transverse, sagittal and dorsal (Figure 20, Figure 21, Figure 22, Figure 23, Figure 24, Figure 25 and Figure 26). This study has been carried out on the leopard, the lion, the cheetah and the cat. 

The nasal bone extends less rostrally in lions than in the other felids. This is seen in Figure 20, where in a rostral cross-section the nasal bone does not cover the roof of the nasal cavity, and again in Figure 25, shown in a sagittal view. Ventrally, the vomer, as well as the palatine process of the maxilla could be clearly observed in the four species in this first level of transverse section (Figure 20). The rostral end of the conchae could not be visualized very well in the leopard or the lion. This fact could also be seen in the sagittal sections (Figure 25B,C). In the cheetah and the cat, the dorsal ventral nasal conchae could be identified as thin, slightly hyperattenuated lamellae. The frontal sinus begins to be observed in the following more caudal sections. 

In a more caudal cross-section, the frontal sinus is visible in the leopard and the cat (Figure 21A,D), but not in the lion or the cheetah. The development of the middle and ventral nasal conchae could already be seen, as well as the great development of the third endoturbinate. Lateral to the ventral nasal concha, the maxillary recess could be identified. The infraorbital foramen was clearly observed at this level in all four species. 

Figure 22 shows the greater thickness of the frontal bone and maxillae of the lion compared to the other species studied. At this level, the temporal process of the zygomatic bone is visible in all four species and the third endoturbinate reaches its greatest development. This was observed as a set of hyperattenuated lamellae on the medial part of the nasal cavity. The ventral nasal concha was already very small in all four species. Ectoturbinates were seen at this level only in the domestic cat. The frontal sinus was now apparent in the cheetah, but not yet in the lion. The tectorial plate of the ethmoid bone was seen as a hyperattenuated area at the base of the frontal sinus.

In the caudal section of the respiratory part of the nasal cavity (Figure 23), the frontal sinus could be observed in the four species. In it, the three ectoturbinates could also be identified as areas limited by hyperattenuated lines, the first and second being more medial and the third developing lateral to these. At this level, the ventral nasal concha could no longer be visualized. We observed that the body of the presphenoid bone closes the caudal wall of the nasal cavity. The space between it and the horizontal and perpendicular plates of the palatine bone forms the choana or the nasopharyngeal opening. The bony borders of the choanae could be seen in the leopard, while in the other species it was no longer ossified.

The last transverse section shows the olfactory part of the nasal cavity (Figure 24). In this section, the ethmoid bone was hyperattenuated, specifically the perpendicular plate, the caudal continuation of the nasal septum. The fourth endoturbinate could now be visualized as a thin hyperattenuated line at this level close to the floor of the nasal cavity.

In the sagittal images of the felid skulls, the bone removal of the ethmoturbinates was clearly observed (Figure 25). Ectoturbinates were observed as a framework of hyperattenuated lines in the frontal sinus. The bony origin of the dorsal and middle nasal conchae and the third and fourth endoturbinates was located in the cribriform plate of the ethmoid bone, which was observed as a thin hyperattenuated vertical line. The ventral nasal concha was observed in the most rostral part; however, in the lion it was not possible to visualize it due to damage to these structures in the fragile skull of this species. The sphenoidal sinus was seen caudal to the fourth endoturbinate, as a single hypoattenuated area.

Finally, in the dorsal section (Figure 26), the ventral nasal concha was visible in the rostrolateral area of the nasal cavity, which extends only to the middle of the cavity; it could be seen laterally in the lion (Figure 26B). The third endoturbinate embraces the ventral nasal concha and occupies most of the cavity. In these sections, the nasal septum was very well identified in the most rostral area and its continuation as a perpendicular plate of the ethmoid bone in the most caudal area of the nasal cavity (Figure 26).

**Figure 27:**
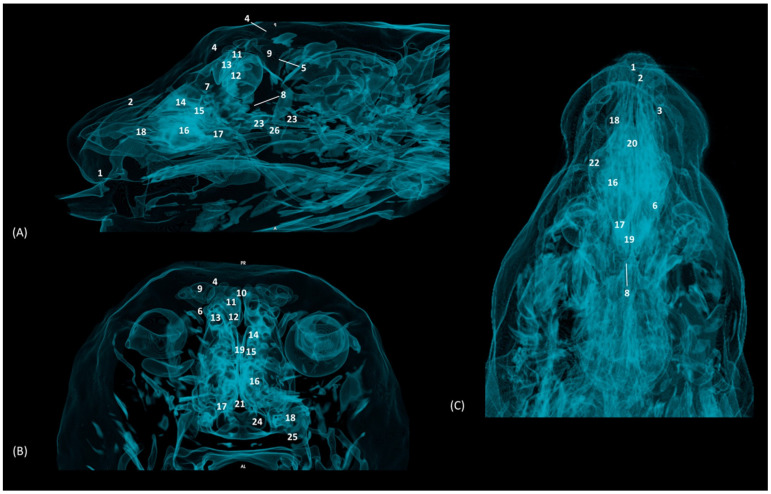
OsiriX MIP reconstructed CT images of the leopard’s head. (**A**) Sagittal image. Left lateral view. (**B**) Transverse image. Caudal view. (**C**) Dorsal image. Dorsal view. 1. Incisive bone; 2. Nasal bone; 3. Maxilla; 4. Frontal bone: external plate; 5. Frontal bone: internal plate; 6. Frontal bone: orbital surface; 7. Ethmoid bone: tectorial plate; 8. Ethmoid bone: cribriform plate; 9. Frontal sinus; 10. Frontal sinus: septum; 11. 1st ectoturbinate; 12. 2nd ectoturbinate; 13. 3rd ectoturbinate; 14. Dorsal nasal concha; 15. Middle nasal concha; 16. 3rd endoturbinate; 17. 4th endoturbinate; 18. Ventral nasal concha; 19. Ethmoid bone: perpendicular plate; 20. Nasal septum; cartilage; 21. Vomer; 22. Zygomatic bone: body; 23. Sphenoidal sinus; 24. Choana; 25. Palatine bone: horizontal and perpendicular plate; 26. Presphenoid bone.

**Figure 28:**
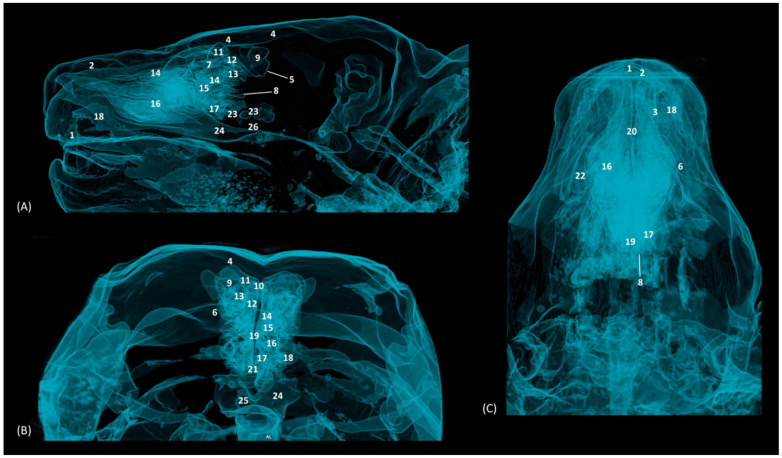
OsiriX MIP reconstructed CT images of the lion’s head. (**A**) Sagittal image. Left lateral view. (**B**) Transverse image. Caudal view. (**C**). Dorsal image. Dorsal view. 1. Incisive bone; 2. Nasal bone; 3. Maxilla; 4. Frontal bone: external plate; 5. Frontal bone: internal plate; 6. Frontal bone: orbital surface; 7. Ethmoid bone: tectorial plate; 8. Ethmoid bone: cribriform plate; 9. Frontal sinus; 10. Frontal sinus: septum; 11. 1st ectoturbinate; 12. 2nd ectoturbinate; 13. 3rd ectoturbinate; 14. Dorsal nasal concha; 15. Middle nasal concha; 16. 3rd endoturbinate; 17. 4th endoturbinate; 18. Ventral nasal concha; 19. Nasal septum: ethmoid bone (perpendicular plate); 20. Nasal septum; cartilage; 21. Vomer; 22. Zygomatic bone: frontal process; 23. Sphenoidal sinus; 24. Choana; 25. Palatine bone: horizontal and perpendicular plate; 26. Presphenoid bone.

**Figure 29:**
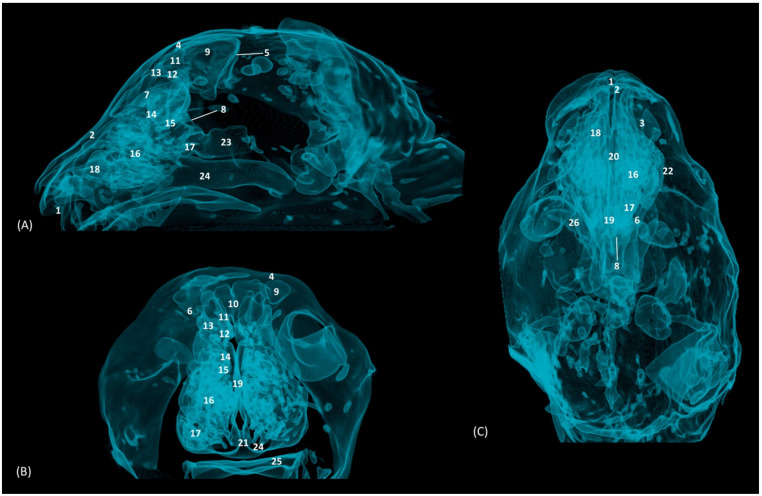
OsiriX MIP CT reconstruction images of the cheetah’s head. (**A**) Sagittal image. Left lateral view. (**B**) Transverse image. Caudal view. (**C**) Dorsal image. Dorsal view. 1. Incisive bone; 2. Nasal bone; 3. Maxilla; 4. Frontal bone: external plate; 5. Frontal bone: internal plate; 6. Frontal bone: orbital surface; 7. Ethmoid bone: tectorial plate; 8. Ethmoid bone: cribriform plate; 9. Frontal sinus; 10. Frontal sinus: septum; 11. 1st ectoturbinate. 12. 2nd ectoturbinate; 13. 3rd ectoturbinate; 14. Dorsal nasal concha; 15. Middle nasal concha; 16. 3rd endoturbinate; 17. 4th endoturbinate; 18. Ventral nasal concha; 19. Nasal septum: ethmoid bone (perpendicular plate); 20. Nasal septum; cartilage; 21. Vomer; 22. Zygomatic bone: frontal process; 23. Sphenoidal sinus; 24. Choana; 25. Palatine bone: horizontal and perpendicular plates; 26. Presphenoid bone.

**Figure 30:**
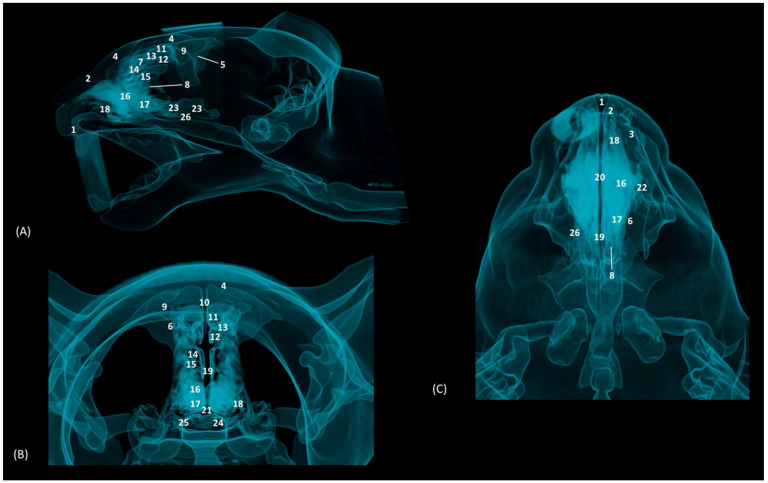
OsiriX MIP reconstruction images of the cat’s head. (**A**) Sagittal image. Left lateral view. (**B**) Transverse image. Caudal view. (**C**) Dorsal image. Dorsal view. 1. Incisive bone; 2. Nasal bone; 3. Maxilla; 4. Frontal bone: external plate; 5. Frontal bone: internal plate; 6. Frontal bone: orbital surface; 7. Ethmoid bone: tectorial plate; 8. Ethmoid bone: cribriform plate; 9. Frontal sinus; 10. Frontal sinus: septum; 11. 1st ectoturbinate. 12. 2nd ectoturbinate; 13. 3rd ectoturbinate; 14. Dorsal nasal concha; 15. Middle nasal concha; 16. 3rd endoturbinate; 17. 4th endoturbinate; 18. Ventral nasal concha; 19. Nasal septum: ethmoid bone (perpendicular plate); 20. Nasal septum: cartilage; 21. Vomer; 22. Zygomatic bone: body; 23. Sphenoidal sinus; 24. Choana; 25. Palatine bone: horizontal and perpendicular plate; 26. Presphenoid bone.

**Figure 31:**
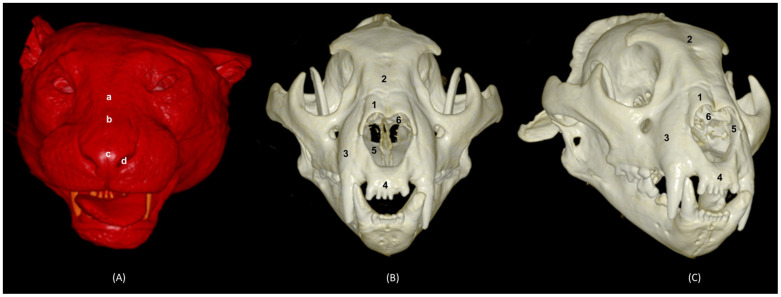
OsiriX 3D reconstructed CT images of the leopard’s head showing the external nares and nasal plane. (**A**): VR surface reconstruction. Rostral view. (**B**): VR deep reconstruction. Rostral view. (**C**) VR deep reconstruction. Right rostrolateral view. a. Root of the nose; b. Dorsum of the nose; c. Tip of the nose; d. Nasal orifice; 1. Nasal bone; 2. Frontal bone; 3. Maxilla; 4. Incisive bone; 5. Nasal cavity; 6. Turbinates.

**Figure 32:**
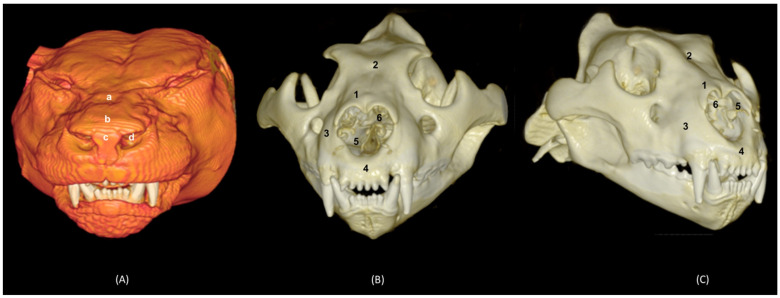
OsiriX 3D reconstructed CT images of the lion’s head showing the external nares and nasal plane. (**A**): VR surface reconstruction. Rostral view. (**B**): VR deep reconstruction. Rostral view. (**C**) VR deep reconstruction. Right rostrolateral view. a. Root of the nose; b. Dorsum of the nose; c. Tip of the nose; d. Nasal orifice; 1. Nasal bone; 2. Frontal bone; 3. Maxilla; 4. Incisive bone; 5. Nasal cavity; 6. Turbinates.

**Figure 33:**
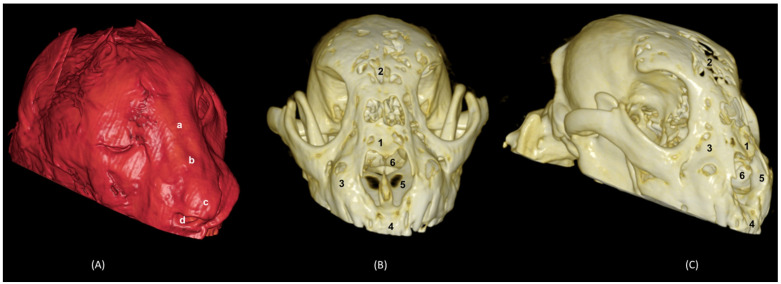
OsiriX 3D reconstructed CT images of the cheetah’s head showing the external nares and nasal plane. (**A**): VR surface reconstruction. Rostral view. (**B**): VR deep reconstruction. Rostral view. (**C**) VR deep reconstruction. Right rostrolateral view. a. Root of the nose; b. Dorsum of the nose; c. Tip of the nose; d. Nasal orifice; 1. Nasal bone; 2. Frontal bone; 3. Maxilla; 4. Incisive bone; 5. Nasal cavity; 6. Turbinates.

**Figure 34:**
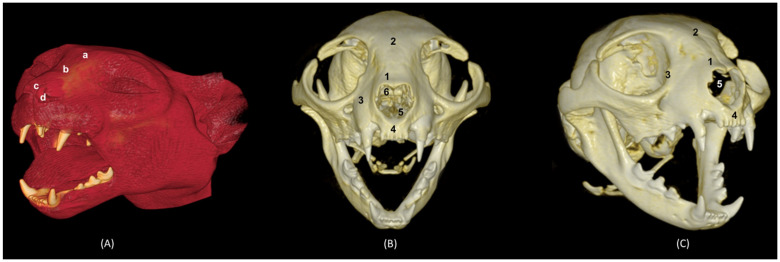
OsiriX 3D reconstructed CT images of the cat’s head showing the external nares and nasal plane. (**A**): VR surface reconstruction. Rostral view. (**B**): VR deep reconstruction. Rostral view. (**C**) VR deep reconstruction. Right rostrolateral view. a. Root of the nose; b. Dorsum of the nose; c. Tip of the nose; d. Nasal orifice; 1. Nasal bone; 2. Frontal bone; 3. Maxilla; 4. Incisive bone; 5. Nasal cavity; 6. Turbinates.

Through tomographic studies carried out on the heads of felids, 3D reconstruction models were developed using 3D MIP (Figure 27, Figure 28, Figure 29 and Figure 30) and VR (Figure 31, Figure 32, Figure 33 and Figure 34) applications. MIP reconstruction techniques allowed us to clearly visualize the internal dimension of each structure of the nasal cavity and paranasal sinuses. Sagittal, transverse, and dorsal views of the four felids were selected.

Starting with the leopard (Figure 27), the extension of the frontal sinus was observed in the sagittal and transverse planes. In this sinus, the very limited ectoturbinates could be visualized, especially in the transverse image. The dorsal nasal concha was observed throughout its entire length, reaching its maximum dimension in the middle area of the nasal cavity and becoming thinner as it approached its rostral end. The ventral nasal concha was limited to the rostral half of the nasal cavity. With this technique, the extension of the choana could also be clearly seen.

Figure 28 represents the MIP reconstruction of the lion’s head. In the sagittal and dorsal planes, the route and dimensions of the ethmoturbinates were observed. The sphenoidal sinus was perfectly located and limited in the sagittal plane, just as the limits of the frontal sinus could be clearly outlined.

The MIP of the cheetah head is represented in Figure 29. The frontal sinus is considerably larger in this species, as is the sphenoidal sinus, which is proportionally much larger than in the other species examined.

Finally, the MIP reconstruction of the domestic cat’s head is shown in Figure 30. The frontal sinus in this species, also proportionally large, deepens to more ventral levels than in the other felids. The ectoturbinates could also be clearly seen in the sagittal and transverse images. Both the path and the width of the nasopharynx (pars nasalis pharyngis) could also be observed extending caudal from the choana.

In addition, four OsiriX surface and deep VR reconstruction CT images of the felid’s heads are presented (Figure 31, Figure 32, Figure 33 and Figure 34). These VR images allowed for the assessment of the external nose and the integrity and the extensions of the bones, enhancing the anatomical understanding of this region.

Figure 31 shows the reconstructed leopard images. The nasal plane and the bony framework that supports the nasal cavity were clearly observed, as well as the boundaries between these bones. In this way, we visualized the frontal and the nasal bones, laterally the maxilla and ventrally the most rostral side of the incisive bone.

In the lion CT VR images (Figure 32), we could also observe the external nose and these bones. However, the morphology of these skulls differed from the rest. The short rostral extension of the nasal bone was observed, as well as the convex morphology that the frontal bone acquires. The bone structure was much more compact than in other felines.

In the reconstruction of the cheetah’s head (Figure 33), the limit between the frontal and nasal bones was not so pronounced. The frontal bone in the cheetah was completely concave, unlike the lion.

Finally, the VR reconstruction of the domestic cat skull (Figure 34) showed that the bone limits were not as noticeable as in the leopard or cheetah. The frontal bone represents a large extension since its zygomatic process developed very laterally, unlike in big felids.

### 3.5. 3D Prints

**Figure 35:**
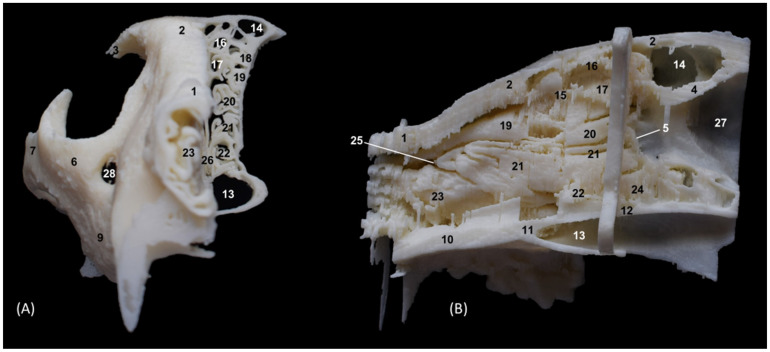
OsiriX 3D printing images of the leopard’s head at the level of the nasal cavity and paranasal sinuses. (**A**) Rostral view so that the dorsal is at the top. (**B**) Left lateral view so that the rostral part is to the left and the dorsal is at the top. 1. Nasal bone; 2. Frontal bone: external plate; 3. Frontal bone: zygomatic process; 4. Frontal bone: internal plate; 5. Ethmoid bone: cribriform plate; 6. Zygomatic bone: body; 7. Zygomatic bone: temporal process; 8. Zygomatic bone: frontal process; 7. Maxilla; 10. Maxilla: palatine process; 11. Palatine bone: perpendicular and horizontal plates; 12. Presphenoid bone: body; 13. Choana; 14. Frontal sinus; 15. Ethmoid bone: tectorial plate; 16. 1st ectoturbinate; 17. 2nd ectoturbinate; 18. 3rd ectoturbinate; 19. Dorsal nasal concha; 20. Middle nasal concha; 21. 3rd endoturbinate; 22. 4th endoturbinate; 23. Ventral nasal concha; 24. Sphenoidal sinus; 25. Maxillary recess; 26. Vomer; 27. Cerebral fossa; 28. Infraorbital canal.

**Figure 36:**
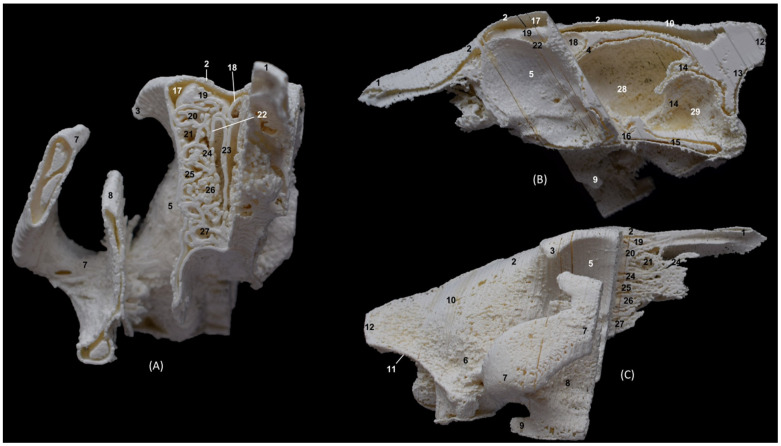
OsiriX 3D printing images of the lion’s head at the level of the nasal cavity and paranasal sinuses. (**A**) Rostral view so that the dorsal is at the top. (**B**) Left lateral view so that the rostral part is to the left and the dorsal is at the top. (**C**) Right lateral view so that the rostral part is to the right and the dorsal is at the top. 1. Nasal bone; 2. Frontal bone: external plate; 3. Frontal bone: zygomatic process; 4. Frontal bone: internal plate; 5. Frontal bone: orbital surface; 6. Temporal bone: squamous part; 7. Temporal bone: zygomatic process; 8. Mandible: ramus; 9. Mandible: angular process; 10. Parietal bone; 11. Occipital bone: nuchal crest; 12. Occipital bone: external occipital protuberance; 13. Occipital bone: squamous part; 14. Osseous tentorium of the cerebellum; 15: Occipital bone: basilar part; 16. Basisphenoid bone: body; 17. Frontal sinus; 18: Frontal sinus: septum; 19. 1st ectoturbinate; 20. 2nd ectoturbinate; 21. 3rd ectoturbinate; 22. Ethmoid bone: tectorial plate; 23. Nasal septum: cartilage; 24. Dorsal nasal concha; 25. Middle nasal concha; 26. 3rd endoturbinate; 27. 4th endoturbinate; 28. Cerebral fossa; 29. Cerebellar fossa.

**Figure 37:**
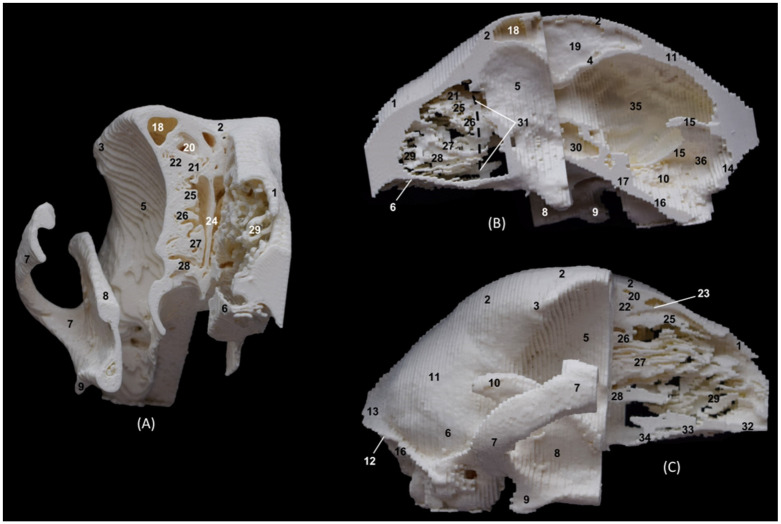
OsiriX 3D printing of the cheetah’s head at the level of the nasal cavity and paranasal sinuses. (**A**) Rostral view so that the dorsal is at the top. (**B**) Left lateral view so that the rostral part is to the left and the dorsal is at the top. (**C**) Right lateral view so that the rostral part is to the right and the dorsal is at the top. 1. Nasal bone; 2. Frontal bone: external plate; 3. Frontal bone: zygomatic process; 4. Frontal bone: internal plate; 5. Frontal bone: orbital surface; 6. Temporal bone: squamous part; 7. Temporal bone: zygomatic process; 8. Mandible: ramus; 9. Mandible: angular process; 10. Mandible: coronoid process; 11. Parietal bone; 12. Occipital bone: nuchal crest; 13. Occipital bone: external occipital protuberance; 14. Occipital bone: squama; 15. Osseous tentorium of the cerebellum; 16: Occipital bone: basilar part; 17. Basisphenoid bone: body; 18. Frontal sinus; 19: Frontal sinus: septum; 20. 1st ectoturbinate; 21. 2nd ectoturbinate; 22. 3rd ectoturbinate; 23. Ethmoid bone: tectorial plate; 24. Nasal septum: cartilage; 25. Dorsal nasal concha; 26. Middle nasal concha; 27. 3rd endoturbinate; 28. 4th endoturbinate; 29. Ventral nasal concha; 30. Sphenoidal sinus; 31. Maxillary recess; 32. Incisive bone: palatine process; 33. Maxilla: palatine process; 34. Palatine bone: horizontal and perpendicular plates; 35. Cerebral fossa; 36. Cerebellar fossa.

**Figure 38:**
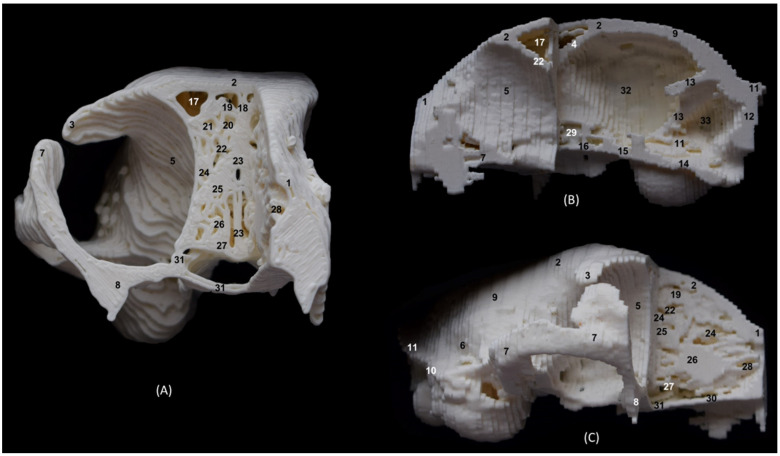
OsiriX 3D printing of the cat’s head at the level of the nasal cavity and paranasal sinuses. (**A**) Rostral view so that the dorsal is at the top. (**B**) Left lateral view so that the rostral part is to the left and the dorsal is at the top. (**C**) Right lateral view so that the rostral part is to the right and the dorsal is at the top. 1. Nasal bone; 2. Frontal bone: external plate; 3. Frontal bone: zygomatic process; 4. Frontal bone: internal plate; 5. Frontal bone: orbital surface; 6. Temporal bone: squamous part; 7. Temporal bone: zygomatic process; 8. Mandible: ramus (sectioned); 9. Parietal bone; 10. Occipital bone: nuchal crest; 11. Occipital bone: external occipital protuberance; 12. Occipital bone: squama; 13. Osseous tentorium of the cerebellum; 14: Occipital bone: basilar part; 15. Basisphenoid bone: body; 16. Presphenoid bone: body; 17. Frontal sinus; 18: Frontal sinus: septum; 19. 1st ectoturbinate; 20. 2nd ectoturbinate; 21. 3rd ectoturbinate; 22. Ethmoid bone: tectorial plate; 23. Nasal septum: cartilage; 24. Dorsal nasal concha; 25. Middle nasal concha; 26. 3rd endoturbinate; 27. 4th endoturbinate; 28. Ventral nasal concha; 29. Sphenoidal sinus; 30. Maxilla: palatine process; 31. Palatine bone: horizontal and perpendicular plates; 32. Cerebral fossa; 33. Cerebellar fossa.

The 3D printing of the feline skulls made it possible to create different molds that were very similar to the anatomy of the live animal (Figure 35, Figure 36, Figure 37 and Figure 38). In this way, we obtained molds of the bony nasal cavity of the leopard, lion, cheetah and domestic cat. These molds were printed with a sagittal section to permit visualization of the entire course of the bone structures in the nasal cavity and a transverse section through the middle of the nasal cavity. The four species studied have different bone densities, and that is why the printed molds of each species had different textures. Figure 35 shows the 3D printing of the leopard’s nasal cavity. In this mold, we observed the three ectoturbinates in the frontal sinus in the transverse plane. On the sagittal view, the sphenoidal sinus could be seen and only the first and second ectoturbinates could be observed since the third develops more laterally. The endoturbinates could be perfectly visualized along their entire length. Furthermore, the rostral view of the nasal cavity presented in the preparation allowed for the observation of the dorsal nasal concha upon arrival at the nasal vestibule, as well as the ventral nasal concha. This only reached the rostral half of the nasal cavity. Except for the ventral nasal concha (in a rostral position), the rest of the endoturbinates were seen as arising from the ethmoid bone, specifically its cribriform plate. Furthermore, with this mold we could also identify the bones that limit the nasal cavity externally, identifying the zygomatic bone and its temporal and frontal processes as well as the zygomatic process of the frontal bone. 

The infraorbital canal was clearly seen.

The 3D printing of the lion’s head (Figure 36) in the transverse plane allowed for the observation of all the ectoturbinates and endoturbinates. In the sagittal plane, the origin of the ectoturbinates and endoturbinates was seen. In addition, the tentorium of the cerebellum was very well ossified and large in size.

The 3D printing of the cheetah’s head (Figure 37) showed, in the same way as mentioned above, a frontal sinus of large proportions and a clear sphenoidal sinus. Furthermore, between the lateral aspect of the ventral nasal concha and the maxilla, the maxillary recess was observed.

Finally, in the bony nasal cavity of the cat (Figure 38), the bony limits of the frontal sinus were very well identified, the roof being represented by the external plate of the frontal bone, the lateral wall by the orbital surface of the frontal bone, the caudal limit by the internal plate of the frontal bone and the floor by the tectorial plate of the ethmoid bone. Both ectoturbinates and endoturbinates could be seen in these molds. It was also observed that the great lateral development of the zygomatic process of the frontal bone, as well as that of the frontal process of the zygomatic bone, were very close to merging, unlike the other species studied. Finally, this 3D printing showed that the ventral nasal concha was embraced medially by the large extension of the third endoturbinate. 

## 4. Discussion

### 4.1. Anatomical Considerations

The exhaustive and detailed knowledge of the anatomy of the bony nasal cavity and paranasal sinuses of the domestic cat, achieved thanks to dissections, trepanations and sectional anatomy techniques, has allowed this species to be considered as a type species, aiding in the study of the nasal cavities of other felids. These techniques have been key to understanding and visualizing many structures that have not yet been identified in felids.

#### 4.1.1. External Nose

The cartilages that make up the external nose are described in the domestic cat using dissection and sectional anatomy. These cartilages, visible after removing the mucosa, have only been described to date in anatomical atlases and texts in the dog [1,29,30], and in the cat and big felids using imaging techniques (CT, MRI and rhinoscopy) by [2] Díaz Martínez et al. [2], but to date its direct visualization as presented in this study has not been published. However, we have not been able to identify the medial accessory nasal cartilage in the cat.

#### 4.1.2. Nasal Vestibule

In the cat, only Barone [30] anatomically described the straight, alar and basal folds. Conchou et al. [11] also identified them using MRI, Losonsky et al. [20] described them using CT, and Díaz Martínez et al. [2] used CT, MRI and rhinoscopy. In the dorsal and sagittal sections of the present study, it was possible to see the extensions of the dorsal and ventral nasal conchae that form the folds in the vestibule in the cat. Neither the MIP nor the VR images showed the folds, but the most rostral part of the turbinates was seen in a rostral view. Likewise, with the 3D printing of the nasal cavity, a mold could be obtained that allowed for the direct visualization of these folds. Thanks to these 3D reconstruction and printing techniques it is possible to obtain very realistic models of the head and nasal cavity of species that, due to their vulnerable state of extinction, such as the leopard, the lion and the cheetah, are more difficult to study.

#### 4.1.3. Nasal Cavity

In this project, the bony nasal cavity and paranasal sinuses were studied in detail with MIP and VR techniques and with subsequent 3D printing to assess the bony limits of the nasal cavity. The lion differs from the other felids in that the frontal bone develops a much more convex morphology. Furthermore, in this species, the zygomatic bone is more developed and extends more laterally. Due to the large size of the parietal and occipital bones, the interior of the lion’s nasal cavity has a greater lateral width and is proportionally lower in height than in other felids. It has also been possible to highlight the greater bone thickness of the lion compared with the other species. A 3D study of the nasal cavity of the leopard, lion and cheetah has only been conducted by Díaz Martínez et al. [2], using rhinoscopy compared with conventional CT and MRI. In the present study, we used CT 3D reconstruction in MIP and VR as well as 3D molds of the heads of big felids and the domestic cat. These techniques allowed us to highlight that in this species, the zygomatic process of the frontal bone is much more developed than in the rest of the felids that we studied. It is indicated that part of the frontal sinus also extends inside it, expanding laterally.

We confirmed that within the bony nasal cavity, the origin of the ethmoturbinates takes place in the cribriform plate of the ethmoid bone, except for the bone of the ventral nasal concha. This concha is fixed to the maxilla, has a feather-shaped morphology, and is topographically located in the rostral half of the nasal cavity. Furthermore, the large extension of the third endoturbinate means that it embraces it and can camouflage it medially depending on the level of section. Barone [30] and Buzek et al. [31] described it in the cat, also highlighting that its extension only reaches the rostral third of the nasal cavity and that it is covered medially by the middle nasal concha. This last concha was also described by Hudson et al. [7]. However, thanks to the dissection and trepanation preparations in this study, we observed how the origin of the middle nasal concha is single, and it is double in the third endoturbinate, and furthermore, this last endoturbinate covers the ventral nasal concha medially. It has also been observed in 3D molds that the bony base of the nasal conchae of the cheetah and the cat are thinner bone sheets than in the leopard and lion. In addition, Crouch [6] also described the bony nasal cavity of the domestic cat, but generically described the ethmoturbinates without differentiating the ectoturbinates, endoturbinates and nasal conchae. The anatomical description of the viscera of the nasal cavity of the cat provided by Konig et al. [8,9] coincides with the results achieved in this study. 

In relation to the paranasal sinuses, MIP and 3D printing images have made it possible to assess the extension of each sinus. Sandoval [1] described that the sphenoidal sinus only differs in bovids and pigs. However, this sinus has been described in cats [7,11] and big felids [2]. The frontal and sphenoidal sinuses in cheetahs and cats reach proportionally large dimensions, unlike those in leopards and in lions. Furthermore, with this MIP technique, it is possible to verify the lateral extension that reaches the frontal sinus in the domestic cat. To date, there are specific studies that describe the paranasal sinuses using imaging techniques and how they are affected in pathologies, but only in dogs [16,32]. Conchou et al. [11] and Díaz Martínez et al. [2] described feline paranasal sinuses using conventional CT and MRI. With the 3D reconstruction techniques (MIP and 3D printing) that we present in this study, the full extent of these sinuses can be seen in a 3D view and, in this way, improve the diagnosis of sinusitis. In addition, the 3D prints allowed for the observation of the extension of the maxillary recess, found only in the cheetah’s head.

CT is a very useful technique for the bony nasal cavity, whose main advantages are the speed of the examination [33] and the ease and clarity of visualizing the bony structures [2]. However, it presents a series of drawbacks that were encountered in our study, such as the difficulty in visualizing very fine and lamellar bone structures, for instance the most rostral parts of the conchae and nasal folds. Addressing the deficiencies that conventional CT presents, the application of VR provides excellent detail of the bony structures of this cavity, improving the interpretation of CT studies [21]. This has allowed for clear images of the bone framework that supports the nasal cavity. The MIP application completes and improves conventional CT. This technique preferably shows bone structures and high-contrast regions [19], a fact that allows for the dimensions of the paranasal sinuses and the path of the ethmoturbinates to be visualized and described in detail.

### 4.2. Clinical Applications

The most common conditions in the nasal cavity of cats are neoplasms, chronic rhinitis, foreign bodies, stenosis in the nasopharynx (pars nasalis pharyngis), nasal polyps, stenotic nasal passages and rhinitis associated with trauma. Cats with neoplasms are highly likely to develop destruction of the nasal turbinates or alterations in the nasal septum or bones that make up the bony framework supporting the nasal cavity [12,13,14,34,35,36]. At the bone level specifically, there are also CT studies describing aberrant nasal turbinates and nasal septum deviations that are more prevalent in brachycephalic dog breeds [37,38], as well as intracranial malformations with or without neurological alterations described in dogs and cats using MRI [39].

There are already some studies that aim to improve the diagnosis of pathologies using these 3D reconstruction techniques. The MIP technique has been used to facilitate the diagnosis in dogs of ectopic ureters, as well as osteoarthrosis in the elbow joint [40,41]. This application has also been used to advance the study of dog anatomy, such as the study of liver vascularization [42]. VR reconstruction has helped to improve understanding in the diagnosis of bone pathologies, such as those affecting the temporomandibular joint in dogs [21]. Longo et al. [43] evaluated the tibial torsion angle in dogs with this technique. Rojo et al. [44] delved into the study of the liver vascularization of the cat, also using these 3D reconstruction techniques, and used 3D prints of the vascularization to provide a more comprehensive study. Furthermore, the combination of MIP and VR techniques improves anatomical studies based on the diagnosis of pathologies [19,45]. To date, 3D studies of the bony nasal cavity of felids or dogs have not been conducted. Bone alterations can be diagnosed in a very graphic way with the 3D techniques shown in our study, and physically they can be captured by creating molds of 3D prints that will improve the understanding and diagnosis of these pathologies [46,47,48].

Regarding the advances published so far on the skull of the domestic cat, research on cats has shown bone morphometric differences of sexual dimorphism in studies on the dimensions of the jaw and pelvis [17]. In addition, morphometric analyses of the skull of the European shorthair cat have been performed using CT. This has allowed for the characterization of the morphometry of the skull of the domestic cat, thus improving the diagnosis and treatment of conditions in this region [18]. Similar studies have also been carried out in dogs [49,50,51]. VR and 3D prints are very useful tools in this area of study that will improve anatomical understanding since they can very precisely capture models that are very realistic. In big felids, there are no studies to date, either morphometric or related to the diagnosis of pathologies at this level. Consequently, these techniques will enhance anatomical studies on these big felids threatened with extinction according to the Red List of Threatened Species of the International Union for Conservation of Nature (IUCN) [52,53,54].

In human medicine, however, 3D representation using VR is already used in the volumetric evaluation of bone defects [25]. 3D printing has already allowed for the creation of molds that facilitate successful surgical planning [25]. Furthermore, this 3D printing of the nasal cavity has already proven to be a suitable option and a rapid method to replicate structures of the nasal cavity, and thus may aid in experimentally testing nasal function [24]. There are also studies that used 3D printing to develop nasal stents in patients after having undergone surgery for cleft lip [28]. In addition, this technique has also allowed for the development of models of the nasal cavity and paranasal sinuses to promote endoscopic training and education in clinicians [27]. Moreover, nasal implants produced by 3D printing in humans have achieved successful results, and the development of molds for craniofacial reconstructions is planned in the same way [26].

These successes achieved in human medicine suggest that these techniques will improve the diagnosis and treatment of pathologies in veterinary medicine. This should have a positive impact on the survival of the species studied, many of them threatened with extinction.

## 5. Conclusions

The bony nasal cavity of the domestic cat has been studied in detail using dissection, trepanation and sectional anatomy techniques. With these techniques, ectoturbinates have been observed with their origin in the cribriform plate of the ethmoid bone and embraced by the tectorial plate. The first and second ectoturbinates develop more medially, and the third remains on a more lateral surface. The dorsal and middle nasal conchae, and the third and fourth endoturbinates also originate from the cribriform plate. However, the ventral nasal concha has a different origin, and instead adheres laterally to the maxilla. Furthermore, this concha, with its feather morphology, differs from the rest of the endoturbinates and has a short extension in felids (and in canids), occupying only the rostral half of the nasal cavity. The third endoturbinate is the most extensive ethmoturbinate and consequently, it has a double origin in the ethmoid bone and largely covers and embraces the ventral nasal concha on its lateral side.

Conventional CT has made it possible to clearly visualize the bony nasal cavity of felids. However, due to the thinness of many of the structures that make up the nasal cavity, the visualization of many of them is difficult. However, through CT 3D reconstruction in MIP and VR, it has been possible to improve and expand the possibilities of conventional CT. In this way, using MIP images, the cavities can be very clearly observed, such as the extension of the paranasal sinuses, as well as the route and dimensions of some nasal conchae. Through MIP reconstruction, it has been possible to assess and study the wide extension that the frontal sinus reaches in the cat and the cheetah compared with other felids. Using VR images, the bony nasal cavity and, above all, the bone framework that supports it can be assessed in detail. Thus, it has been possible to identify the different conformations of the frontal bone of the lion with its convex development as well as the short extension that the nasal bone reaches in this species. In addition, it has also been possible to observe the great extension of the zygomatic process of the frontal bone in cats, laterally expanding the frontal sinus. In the domestic cat, the development of the frontal process of the zygomatic bone is very pronounced, unlike other felids.

Finally, 3D printing of the bony nasal cavity has made it possible to obtain molds of great precision and anatomical detail. This is a key tool to improving the anatomical study of the nasal cavity of these species, especially for those whose vulnerable state of extinction makes it more difficult to obtain specimens for study. Furthermore, in humans, 3D printing already has successful clinical applications that are improving the diagnosis and treatment of pathologies. In veterinary medicine, although it has not yet been fully implemented, it represents a very promising advance in the clinical field that will also improve the diagnosis of several pathologies at this level and help with surgical planning, as well as the generation of surgical implants that can improve the survival of many exotic species.

## Data Availability

The information can be requested from elena.diaz@mivet.com and ricardo.n.l@um.es.
